# Supplementary material for: Active and conductive layer stacked superlattices for highly selective CO2 electroreduction
Source: Nat Commun. 2022 Apr 19;13:2039. doi: 10.1038/s41467-022-29699-2 (PMC9018841; doi:10.1038/s41467-022-29699-2)
Supplement: Supplementary file 1 — Supplementary Information [file 41467_2022_29699_MOESM1_ESM.pdf]

Supplementary Information

Active and Conductive Layer Stacked Superlattices for Highly Selective CO_2 Electroreduction

Junyuan Duan[1], Tianyang Liu[2], Yinghe Zhao[1], Ruoou Yang[1], Yang Zhao[1], Wenbin Wang[1], Youwen Liu[1]*, Huiqiao Li[1], Yafei Li[2]* & Tianyou Zhai[1]*

[1]State Key Laboratory of Materials Processing and Die & Mould Technology, and School of Materials Science and Engineering, Huazhong University of Science and Technology, Wuhan, Hubei, 430074, P. R. China.

[2]Jiangsu Collaborative Innovation Centre of Biomedical Functional Materials, Jiangsu Key Laboratory of New Power Batteries, School of Chemistry and Materials Science, Nanjing Normal University, Nanjing, Jiangsu, 210023, P. R. China

E-mail: zhaity@hust.edu.cn; ywliu@hust.edu.cn; liyafei@njnu.edu.cn

S1. Structural characterizations and formation energy of BiCuSeO

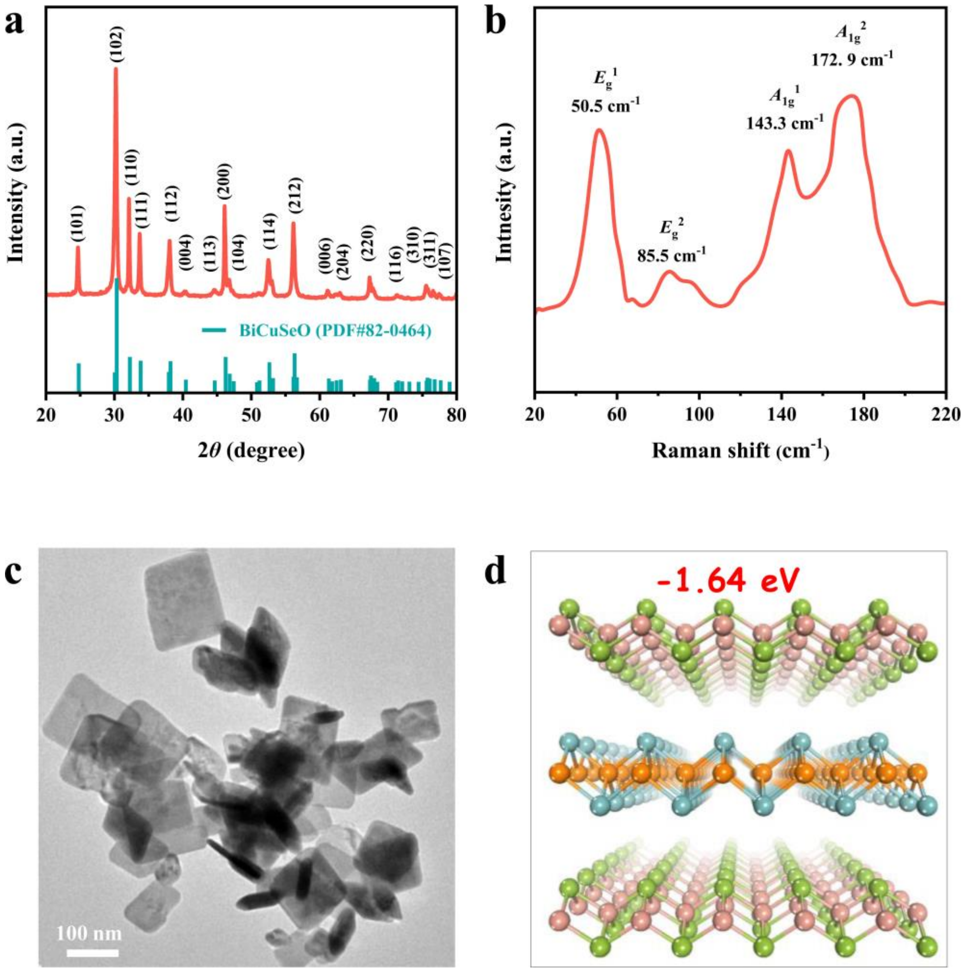

Supplementary Fig. S1 (a) XRD pattern, (b) Raman spectrum, and (c) TEM image of ultrathin BiCuSeO nanosheets. (d) The formation energy of $[Bi_2O_2]^{2+}$ sublayer in BiCuSeO system. All diffraction peaks in XRD pattern (Supplementary Fig. S1a) can be assigned to the tetragonal BiCuSeO phase with the P4/nmm space group (JCPDS card No. 82-0464)[1, 2, 3], and no other impurity peaks are discernable, indicating that the crystalline BiCuSeO single phase is successfully obtained. Moreover, two-humped peaks located at 50.5 (E_g^1) and 85.5 cm^{-1} (E_g^2) corresponding to the in-plane motion of Cu atoms, and peaks at 143.3 (A_{1g}^1) and 179.2 cm^{-1} (A_{1g}^2) corresponding to the out-of-plane vibration of Bi and Se atoms in the tetrahedral can be clearly observed from Raman spectrum (Supplementary Fig. S1b)[4], further illustrating the successful preparation of BiCuSeO nanosheets. The calculation result in Supplementary Fig. S1d shows that the formation energy of $[Bi_2O_2]^{2+}$ in BiCuSeO system is -1.64 eV, suggesting the easy formation of $[Bi_2O_2]^{2+}$ and a high stability of BiCuSeO during the reaction. The experimental and theoretical calculation results consistently indicate that crystalline BiCuSeO with high purity and good stability has been successfully synthesized.

S2. Schematic diagram of flow cell

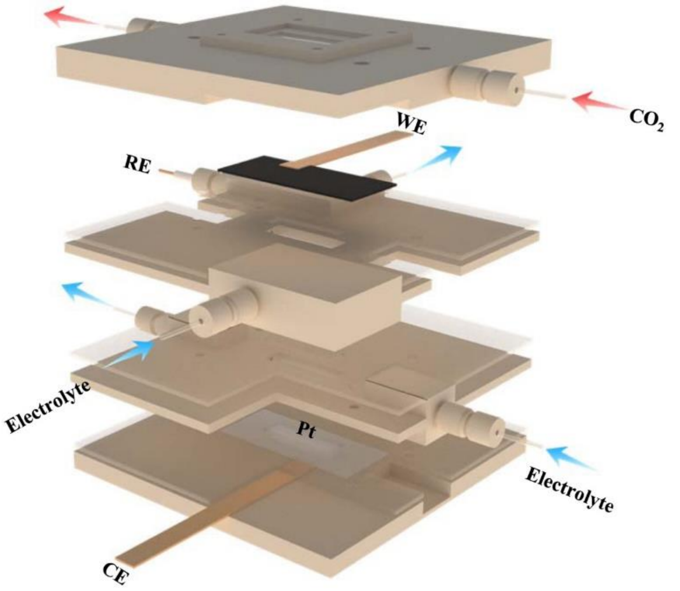

Supplementary Fig. S2 The schematic diagram of the three-electrode flow cell system. The working electrode (WE) is a catalyst-deposited on gas diffusion layer of working area in a 1 cm^2. The reference electrode (RE) and counter electrode (CE) are Ag/AgCl and Pt plate, respectively. CO_2 gas is continually supplied by a steady flow pump with a flow rate of 20 mL min^{-1}. Electrolyte is correspondingly pumped into anode and cathode cell by a peristaltic pump.

S3. Structural characterizations of Cu_2Se Ns

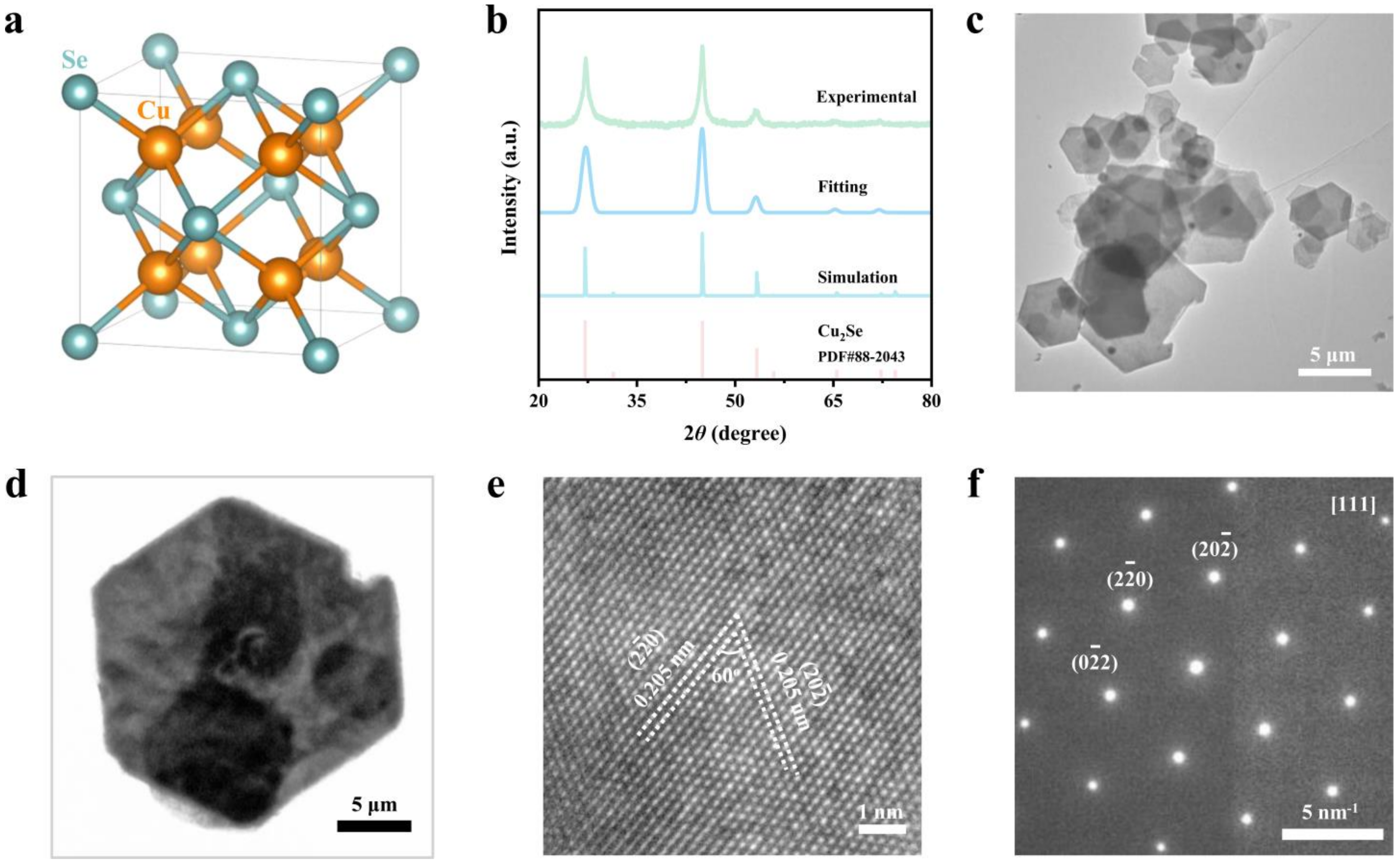

Supplementary Fig. S3 (a) Structure model, (b) XRD pattern, (c, d) TEM images, (e) HRTEM image and (d) SAED of the as-prepared Cu_2Se nanosheets. XRD pattern exhibits the only diffraction peaks of cubic phase Cu_2Se, which is consistent with the reported data (JCPDS No. 88-2044)[5]. Sharp and intense peaks suggest their highly crystalline nature. TEM images shows that the Cu_2Se samples are uniform hexagonal nanosheets. HRTEM image and SAED pattern illustrate that the as-obtained Cu_2Se is single crystal. Taken together, the above results indicate that uniform and dispersible Cu_2Se single crystalline hexagonal nanosheets of high purity are obtained.

S4. Structural characterizations of Bi_2O_3 Ns

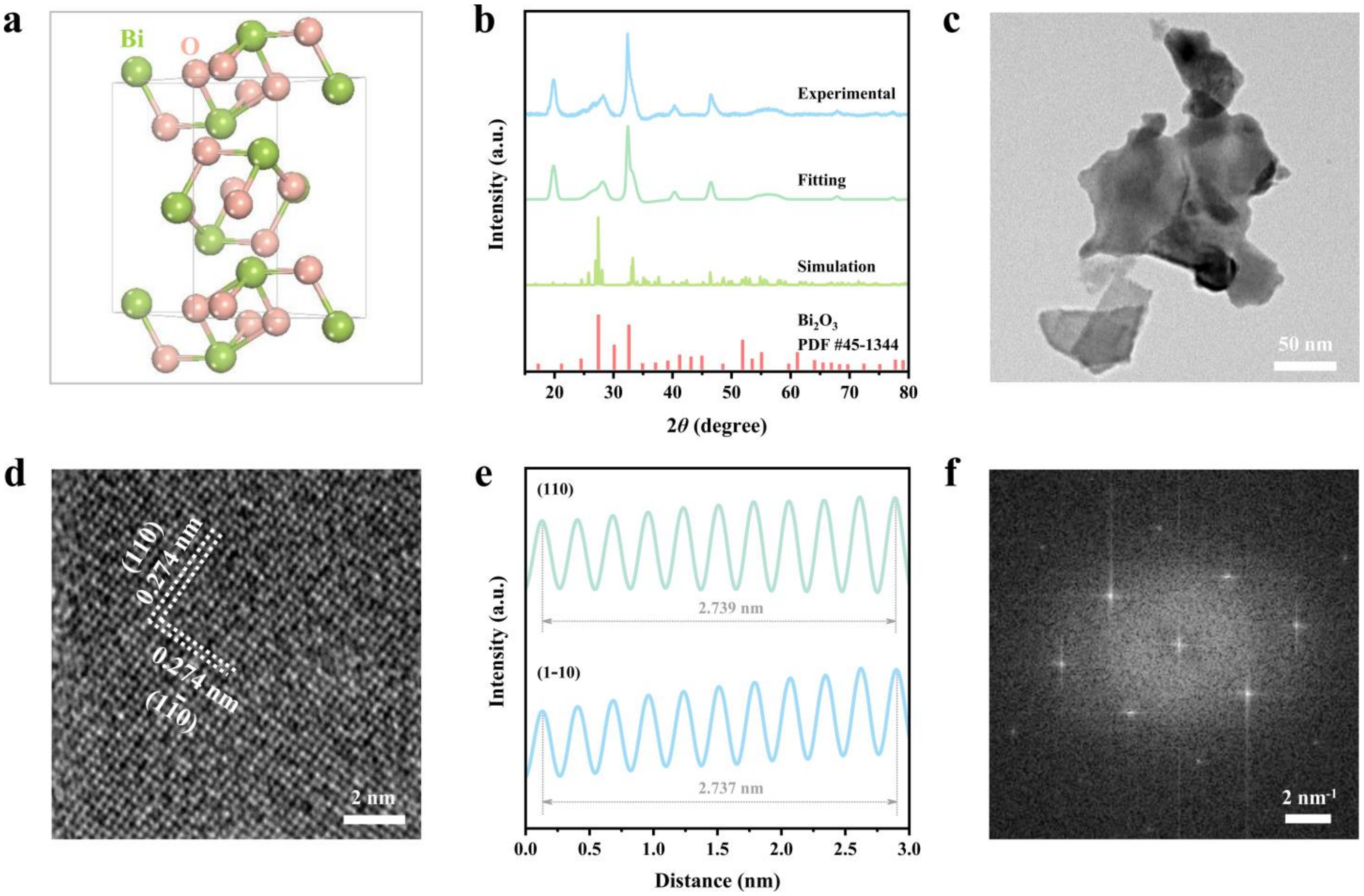

Supplementary Fig. S4 (a) Structure model, (b) XRD pattern, (c) TEM image, (d) HRTEM image and (d) SAED of the as-prepared Bi_2O_3 nanosheets. (e) The interplanar spacings of the lattice fringes. (f) FFT image of Supplementary Fig. S5d. XRD pattern show crystalline Bi_2O_3 is obtained. TEM image reveals the obvious nanosheet-liked shape. HRTEM and FFT images illustrates the its single-crystal nature. The characterization results in Supplementary Fig. S4 show that single-crystalline Bi_2O_3 nanosheets are successfully prepared.

S5. Structural characterizations of CuSe-BiO heterostructures

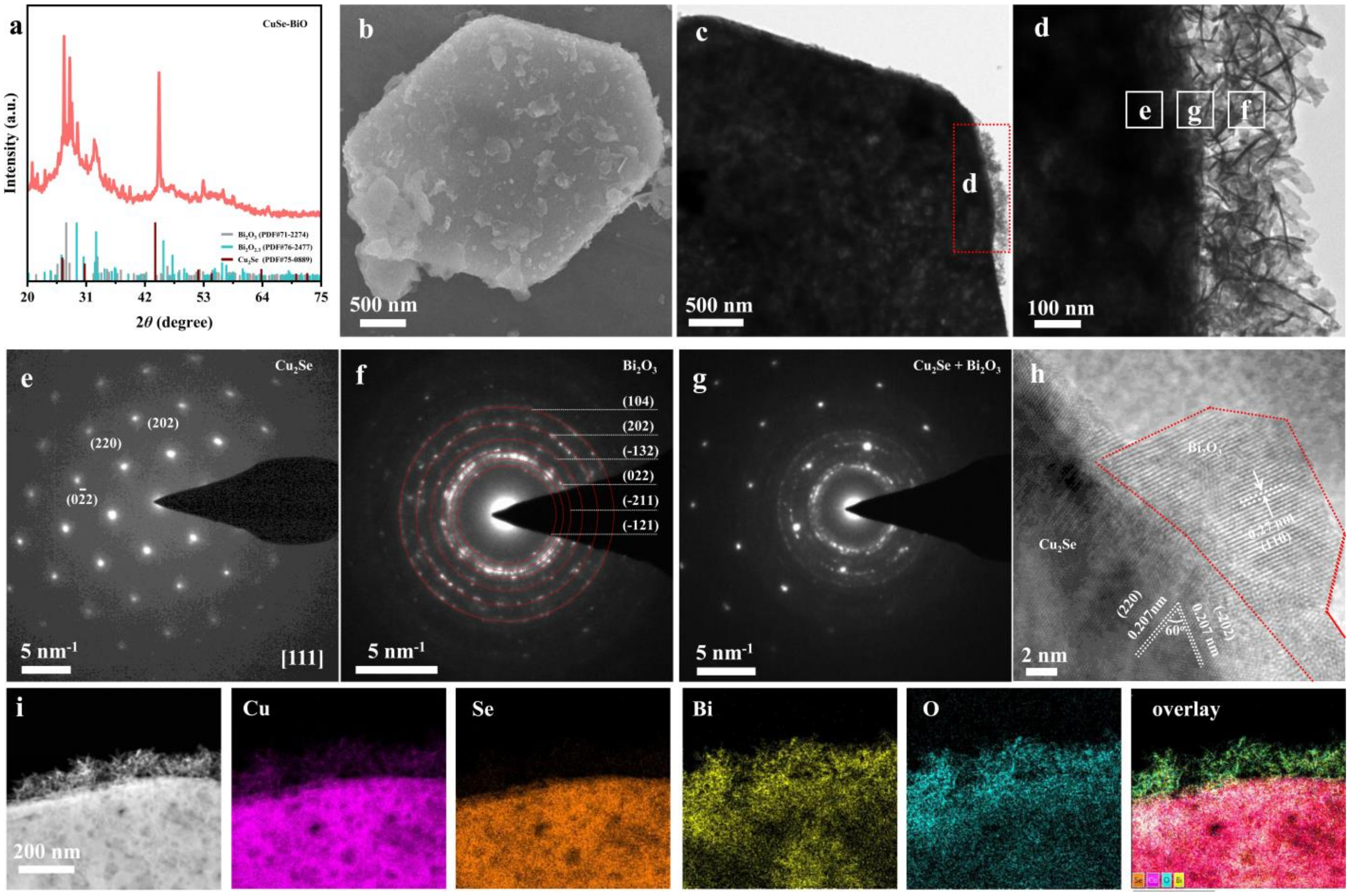

Supplementary Fig. S5 Structural characterizations of Cu_2Se/Bi_2O_3 hetero-structures (CuSe-BiO). (a) XRD pattern. (b) SEM image. (c, d) TEM images. (e, f, g) Corresponding SAED patterns of the areas indicated with the rectangular frame e, f, g respectively. (h) HRTEM image. (i) EDS mapping. Both the diffraction peaks corresponding to cubic phase Cu_2Se and Bi_2O_3 appear in the XRD pattern (Supplementary Fig. S5a), suggesting the successful synthetization of hetero-structures including crystalline Cu_2Se and Bi_2O_3. SEM and TEM images clearly show that a lot of small nanosheets in irregular morphologies are grown on the large-size hexagonal sheets. Compare with the pristine Cu_2Se nanosheets (Supplementary Fig. S3) and the previously synthesized Bi_2O_3 nanosheets (Supplementary Fig. S4), it can be deduced that the large sheet is Cu_2Se and the small ones are Bi_2O_3. SAED patterns taken at the areas of e and f exhibit a set of single crystal diffraction spots matched to Cu_2Se phase and polycrystalline electron diffraction rings matched to Bi_2O_3 phase respectively. Notably, both the single crystal diffraction spots corresponding to Cu_2Se phase and polycrystalline diffraction rings corresponding to Bi_2O_3 phase appeared simultaneously in SAED pattern taken at the interface area. Carefully observing HRTEM image, the area of the large nanosheets displayed lattice fringe distance of 0.207 nm, according with the (2-20) and (20-2) plane of cubic Cu_2Se (Supplementary Fig. S5h). By contrast, the lattice spacing of 0.27 nm was observed on the small nanosheet grown on the large sheet (Supplementary Fig. S5h), which was consistent with the (110) plane of Bi_2O_3. In addition, EDS element mapping image showed that Cu and Se elements were only distributed in the large Cu_2Se nanosheets, while Bi and O elements were distributed on the external Bi_2O_3 nanosheets (Supplementary Fig. S5i). Taken together, heterostructures with Bi_2O_3 nanosheets grown Cu_2Se sheet (CuSe-BiO) were successfully prepared.

S6. ^{1}H NMR spectra of the liquid product at different potential

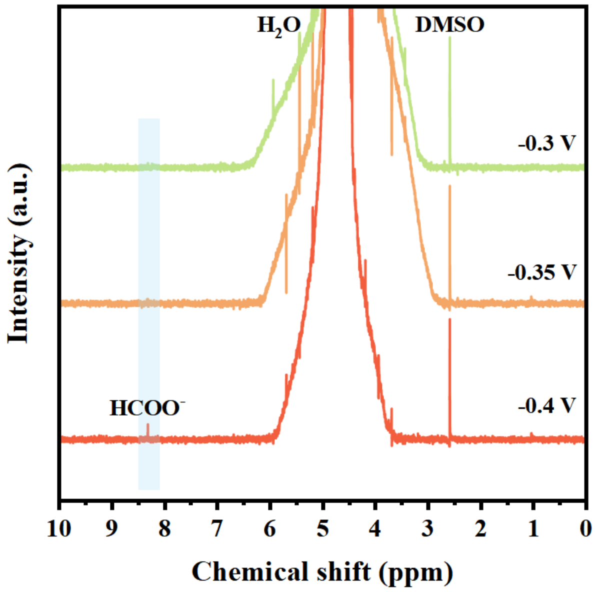

Supplementary Fig. S6 ^{1}H NMR spectra of the liquid product at different potential. The NMR spectra clearly showed that formate could be produced at -0.4 V. Besides, weak signal of formate seemed to be occurred at the NMR spectrum of liquid product obtained at -0.35 V. This result displayed that formate could be generated at a relatively potential of -0.4 V.

S7. CO_2RR performance BiCuSeO in 0.5M $KHCO_3$ solution with iR compensation

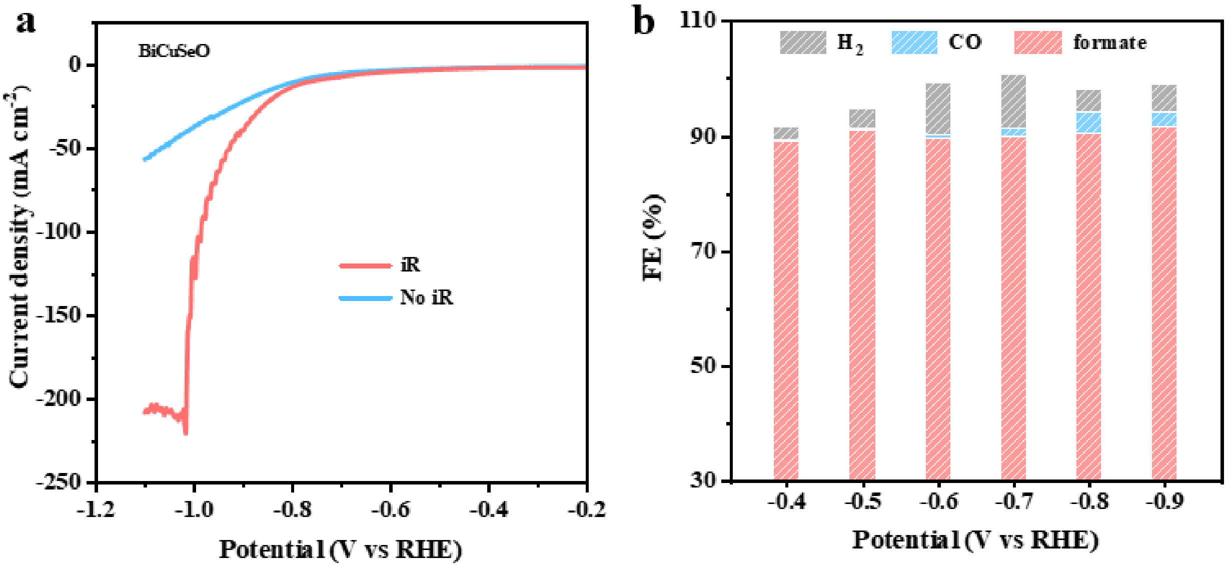

Supplementary Fig. S7 CO_2RR performance of BiCuSeO in 0.5 M $KHCO_3$. (a) LSV curves with and without iR compensation. (b) $FE_{formate}$ with iR compensation. When BiCuSeO catalyst is measured with iR compensation in 0.5 M $KHCO_3$ solution, the LSV curve clearly exhibits the significantly larger current density in compared with that tested without iR compensation. The maximum current density reaches to ~ 219 mA cm^{-2} (Supplementary Fig. S7a), which is ~3.86 times of that gained without iR compensation. Moreover, BiCuSeO also maintains an outstanding formate selectivity over a wide potential window with iR compensation (Supplementary Fig. S7b).

S8. Electrochemical measurements of BiCuSeO in CO_2-saturated 1M KOH solution

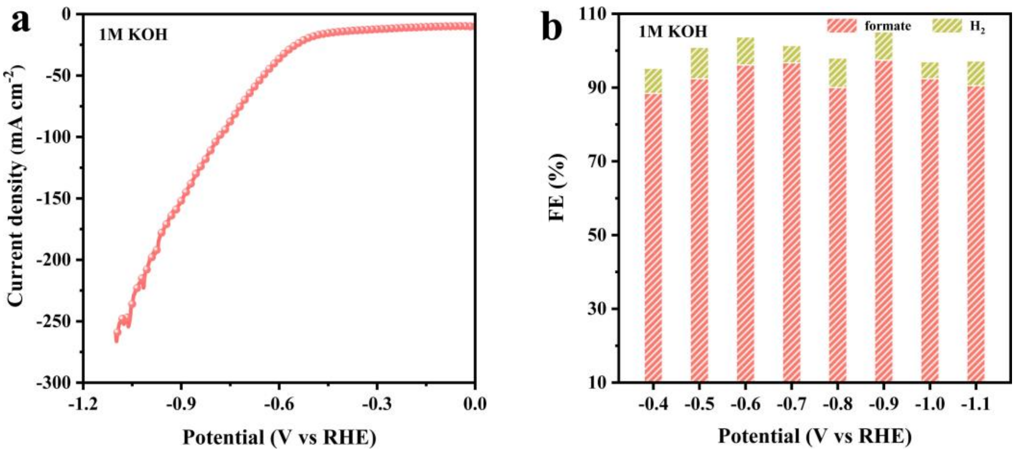

Supplementary Fig. S8 (a) LSV curve. (b) Potential-dependent Faradaic efficiency (FE) of formate and H_2. When BiCuSeO catalyst is measured in CO_2-saturated 1M KOH solution, the LSV curve clearly manifests the significantly larger current density in compared with that tested in $KHCO_3$ solution. The current density reaches to ~267 mA cm^{-2} at -1.1 V, which is 4.63 times of that gained in $KHCO_3$ solution. The formate selectivity in 1M KOH is also satisfactory, and measured to be ~90% in wide potential window (from -0.4 to -1.1 V).

S9. CV curves with different scan rates

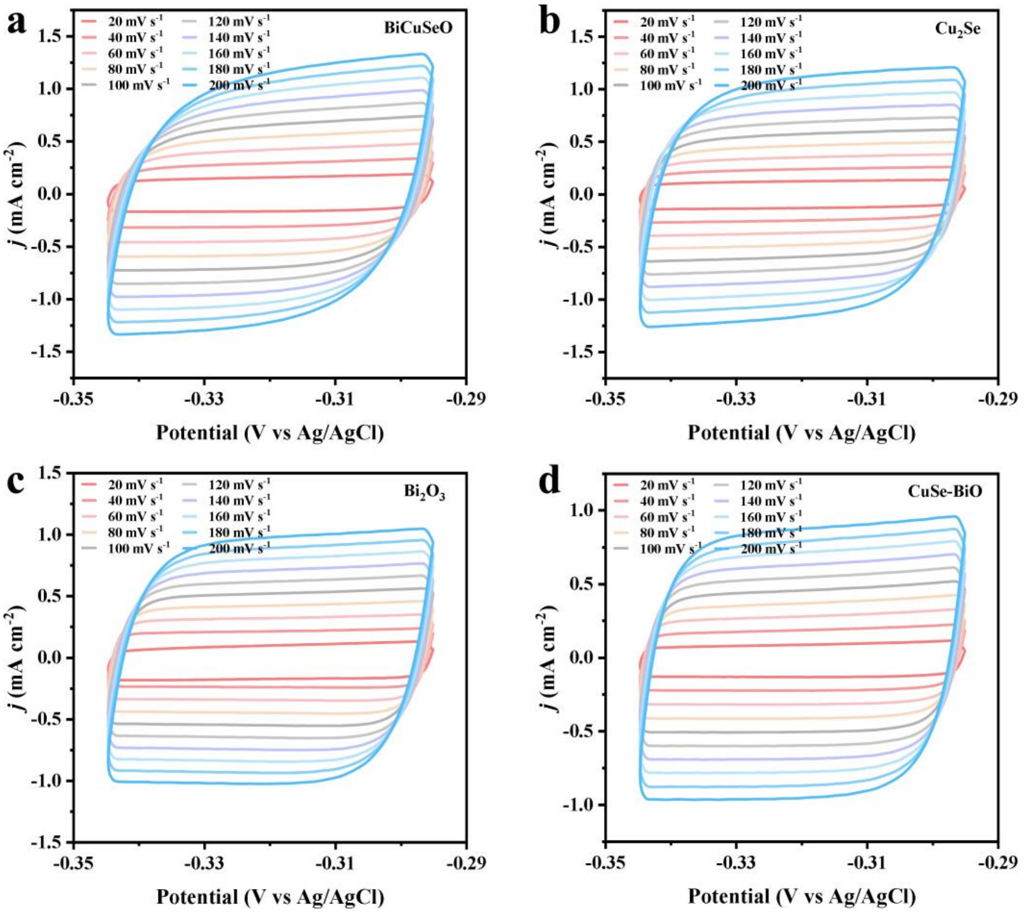

Supplementary Fig. S9 CV curves with different scan rates. (a) BiCuSeO, (b) Cu_2Se, (c) Bi_2O_3. (d) CuSe-BiO.

S10. C_{dl} amd ECSA-normalized formate current densities

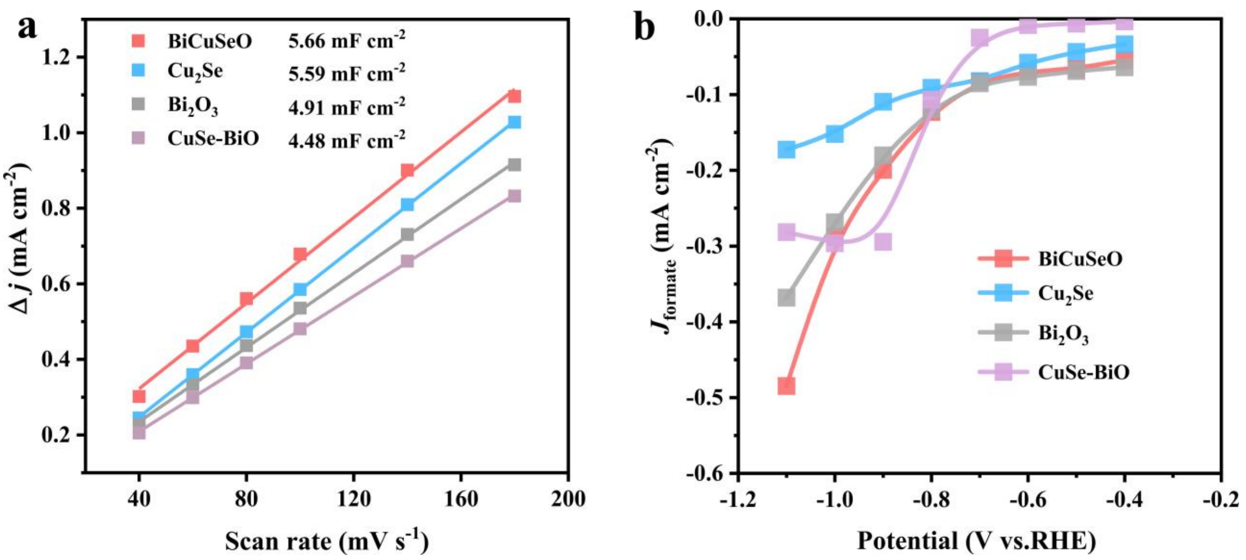

Supplementary Fig. S10 (a) C_{dl} comparisons by dividing the double layer charging current differences with the scan rates. (b) ECSA-normalized formate current densities. The electrochemical double electric layer capacitances (C_{dl}) of BiCuSeO, Cu_2Se, Bi_2O_3 and CuSe-BiO were calculated to be 5.66, 5.59, 4.91 and 4.48 mF cm^{-2} respectively by using the equation $C_{dl} = \Delta j/v$, where Δj and v were current density difference and scan rates respectively. Accordingly, the ESCA values of BiCuSeO, Cu_2Se, Bi_2O_3 and CuSe-BiO were calculated to be 94.3. 93.2, 81.8, and 74.7 according to the formula ESCA = C_{dl}/C_s (C_s = 60 μF cm^{-2}). Clearly, BiCuSeO exhibited the largest ESCA. Even more, the BiCuSeO still showed the maximum and even greatly increased current density at all applied potentials, suggesting a large increase of intrinsic activities refer to Bi_2O_3, Cu_2Se and CuSe-BiO.

S11. SEM images of BiCuSeO and $BiCuSeO_R$

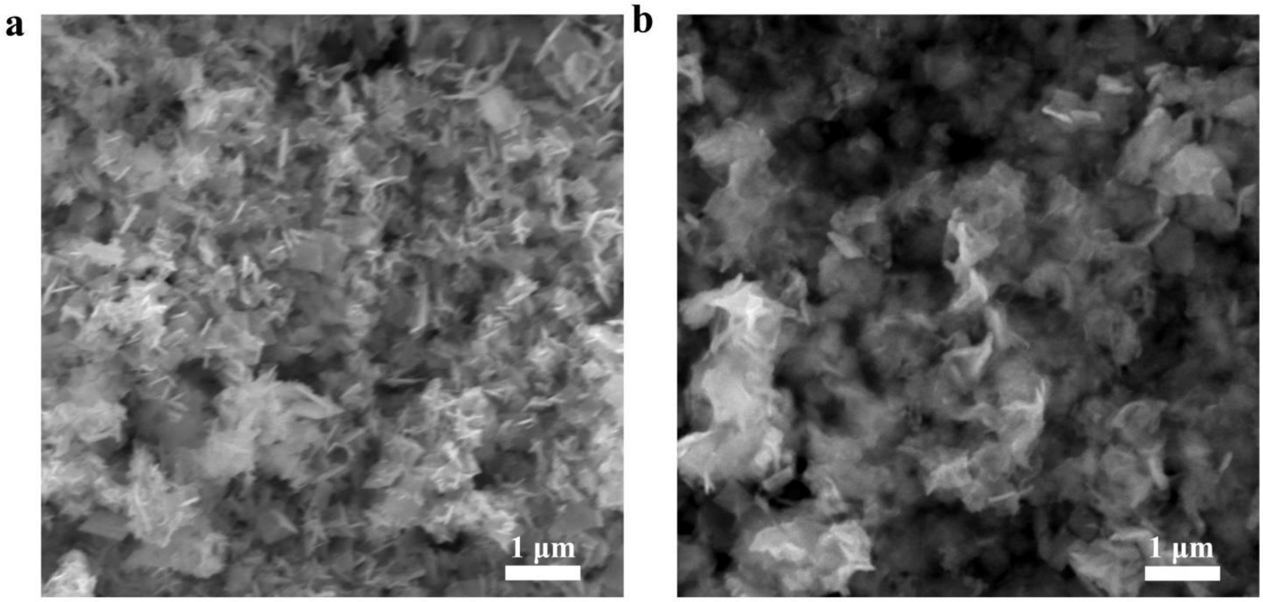

Supplementary Fig. S11 (a) Pristine BiCuSeO samples. (b) $BiCuSeO_R$ samples collected after 10 h electrochemical measurements for CO_2RR. It can be clearly observed from SEM images that $BiCuSeO_R$ after 10 h CO_2RR testing exhibits a similar nanosheet-liked morphology and size as pristine BiCuSeO, suggesting both of them are relatively stable during the electro-catalytic CO_2RR process.

S12. Computed Raman vibrational features for the intermediates

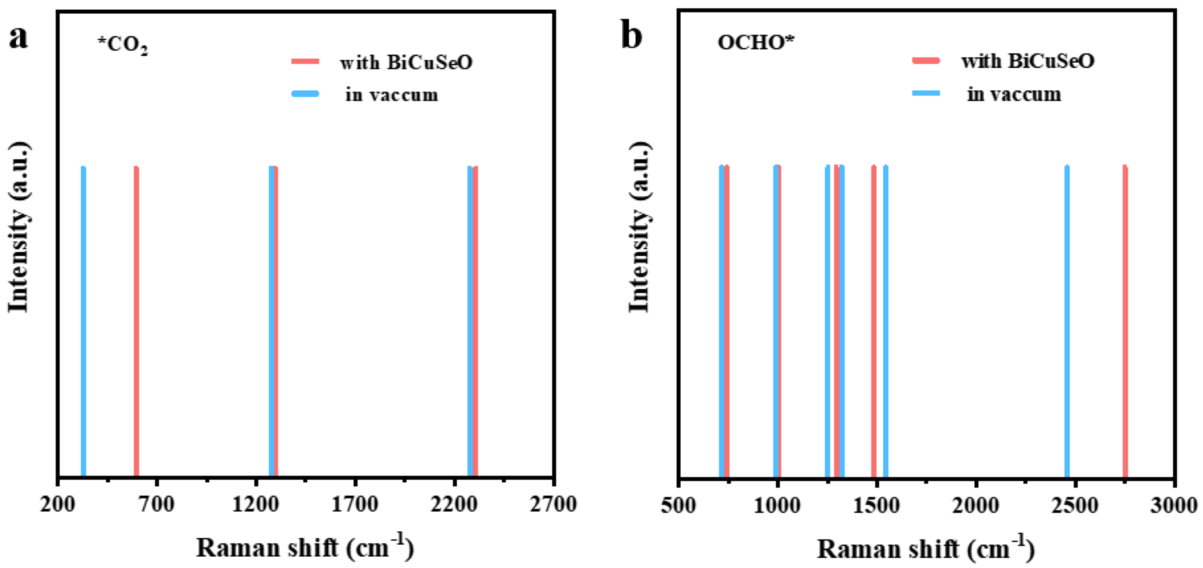

Supplementary Fig. S12 Computed Raman vibrational features for the intermediates (a) $*CO_2$ and (b) OCHO*. The calculated Raman peaks of asymmetric C=O stretching vibration modes for OCHO* is located at 1544.98 cm^{-1} (Table S6 and Supplementary Fig. S12b), which agreed well the experimental Raman measurement (1540 cm^{-1}). The calculated Raman vibration peak of $*CO_2$ was located at 1295.1 cm^{-1}, which can be corresponded to the experimental test of $*CO_2$ at 1160 cm^{-1} (Supplementary Table S5 and Fig. S12a). The difference between theoretical and experimental values might be caused by the electrolyte environment and complex structure vibration of CO_2 molecules.

S13. Schematic illustration of reaction mechanism for the CO_2RR to formate

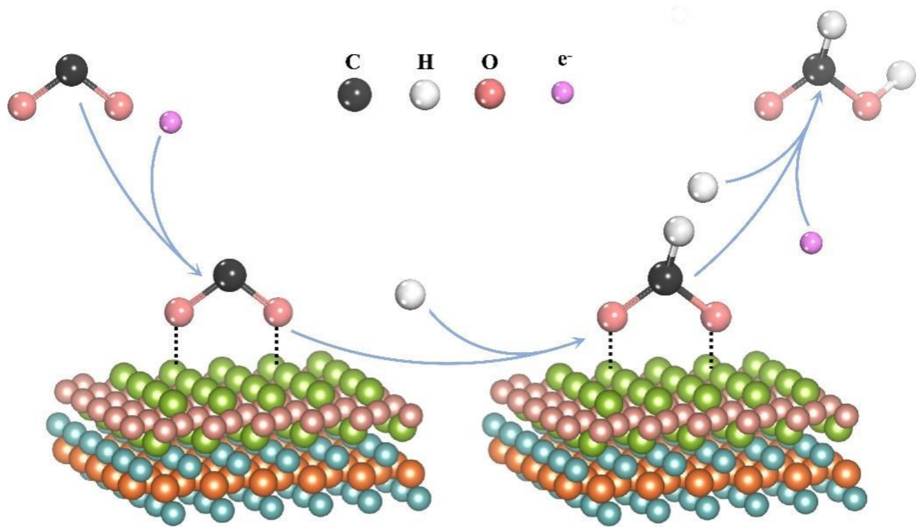

Supplementary Fig. S13 Schematic illustration of reaction mechanism for the CO_2RR to formate from in situ Raman spectroscopy. Raman peaks of $\nu_s CO_2^{\bullet -}$ and $\nu_{as} CO_2^{\bullet -}$ respectively corresponding to two key intermediates of formate formation $*CO_2^{\bullet -}$ and HCOO*can be detected by Raman spectroscopy virtually[6, 7]. And, the reaction mechanism for CO_2RR to formate can be proposed. Firstly, one electron transfers to surface-adsorbed CO_2 molecule to form first intermediate $*CO_2^{\bullet -}$ ($CO_2 + e^- = *CO_2^-$). Subsequently the $*CO_2^{\bullet -}$ is changed into OCHO* by coupling a proton through its C atom ($*CO_2^- + H^+ =$ OCHO*). Further one electron and proton, HCOO*can be directed toward formate (OCHO* + $H^+ + e^-$ = HCOOH).

S14. Structural characterizations of $BiCuSeO_R$ samples collected after CO_2RR

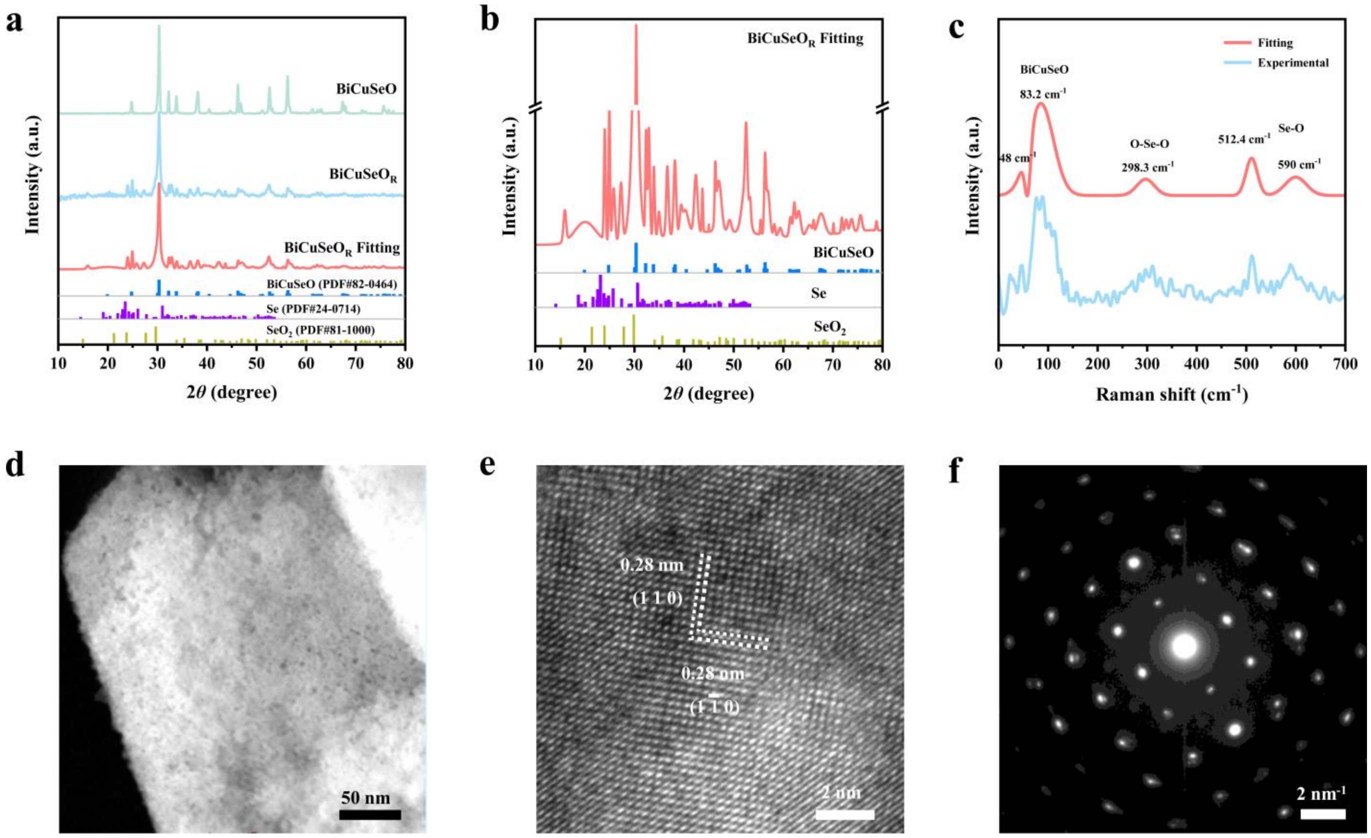

Supplementary Fig. S14 (a) Comparisons of XRD patterns for pristine BiCuSeO, experimental $BiCuSeO_R$ and fitting $BiCuSeO_R$. (b) The corresponding enlarged XRD pattern of $BiCuSeO_R$ after fitting. (c) Raman spectrum of experimental and fitting $BiCuSeO_R$. The XRD pattern as-shown in Supplementary Fig. S9a visibly displays that the dominant phase in $BiCuSeO_R$ samples is composed of the tetragonal BiCuSeO phase (JCPDS card No. 82-0464). This result illustrates the BiCuSeO phase can be well retained during CO_2RR process. Further enlarging its XRD pattern, a small quantity of Se and SeO_2 phase are also observed, reasonably suggesting that Se atoms are escaped from Cu_2Se_2 layers through the interlayer space during electrochemical reaction process. Noteworthy, besides the peak at 83.2 cm^{-1} corresponding to the in-plane motion of Cu atoms of BiCuSeO[4], peaks at 298.3, 512.4 and 512.4 cm^{-1} respectively corresponding to O-Se-O bending vibration mode (E_g), Se-O stretching vibration mode (E_g) and Se-O stretching vibration modes (A_{1g},) of solid SeO_2 are examined in the Raman spectrum of $BiCuSeO_R$ sample[8], further confirming the reservations of BiCuSeO phase and simultaneous escaping of Se atoms. Moreover, TEM image in Supplementary Fig. S9d shows that the sheet-liked morphology remains essentially unchanged, which is good consistent with the SEM images. Close observation displays that the nanosheets contain numerous pores, which is probably ascribed to the escaping of Se atoms. Mutually perpendicular lattice fringe appearing in HRTEM and a single set of diffraction spots presenting in SAED demonstrate its single-crystalline nature. Taken together, the above results suggest that the geometric structure of BiCuSeO can be topologically maintained during CO_2RR process.

S15. *q* space curves of Fourier-transform EXAFS spectra

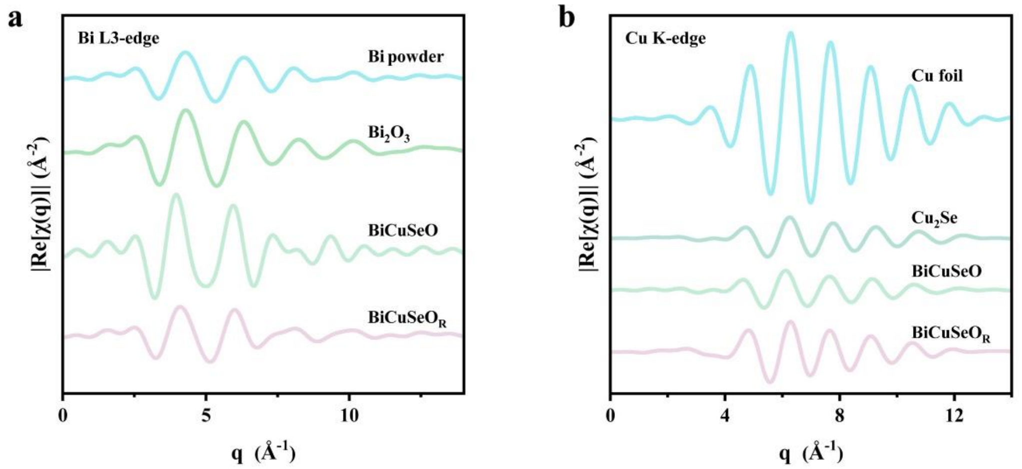

Supplementary Fig. S15 *q* space curves of (a) Bi L3-edge and (b) Cu K-edge FT-EXAFS for BiCuSeO, $BiCuSeO_R$ and reference samples.

S16. XANES spectrum and linear combination fitting results of $BiCuSeO_R$

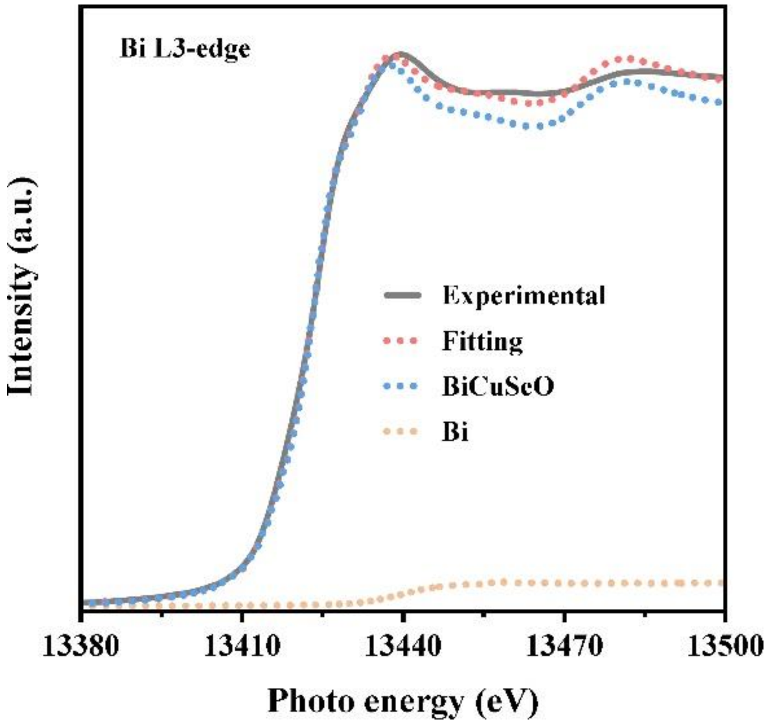

Supplementary Fig. S16 XANES spectrum and linear combination fitting results of $BiCuSeO_R$. According to the linear combination fitting of XANES spectrum of $BiCuSeO_R$, it can be found that the content of metal Bi is less than 5% (~4.4%), indicating that the oxidation state of Bi mainly is retained in after $BiCuSeO_R$ CO_2RR.

S17. EXAFS R and k space fitting results

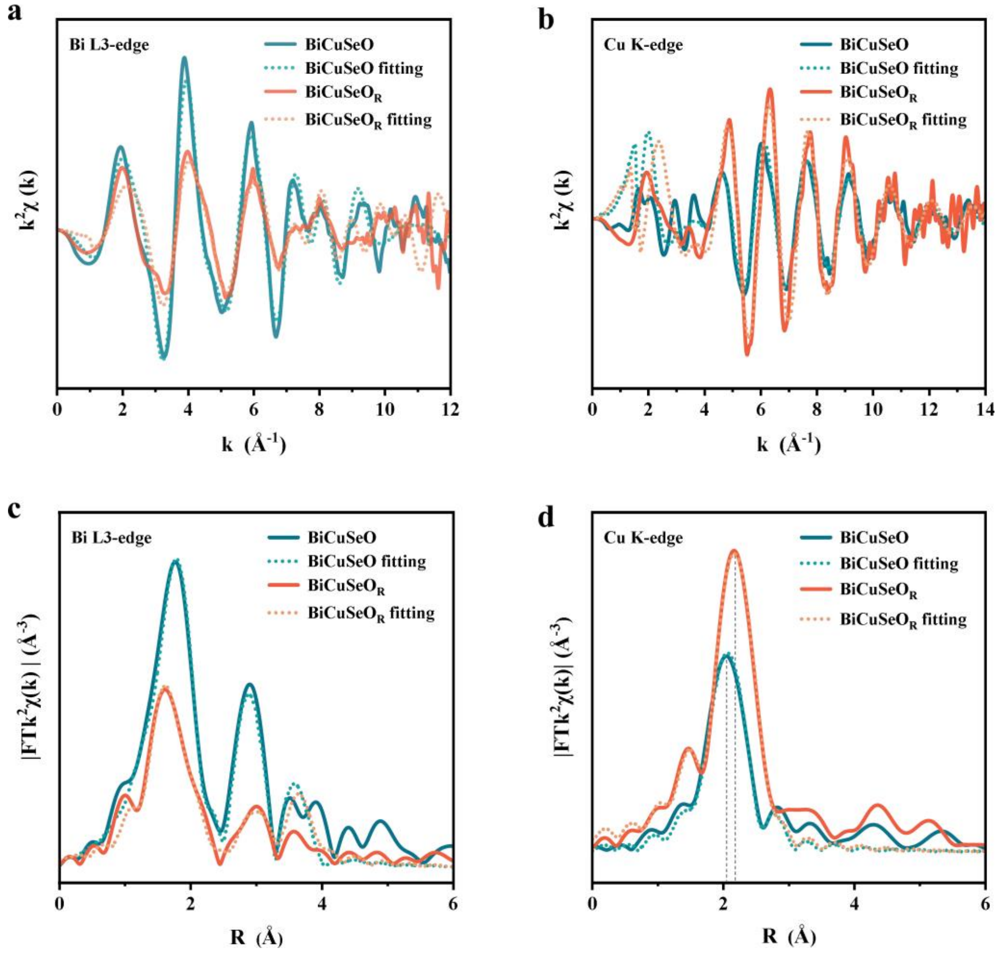

Supplementary Fig. S17 Experimental and fitting data of $k^2\chi(k)$ oscillation functions curves for (a) Bi L3-edge and (b) Cu K-edge k space spectra. The corresponding Fourier-transform curves for (c) Bi L3-edge and (d) Cu K-edge EXAFS spectra. A good fitting quality with the curve fitting in k and R spaces can be obtained from Supplementary Fig. S17.

S18. Bi L3-edge EXAFS fitting

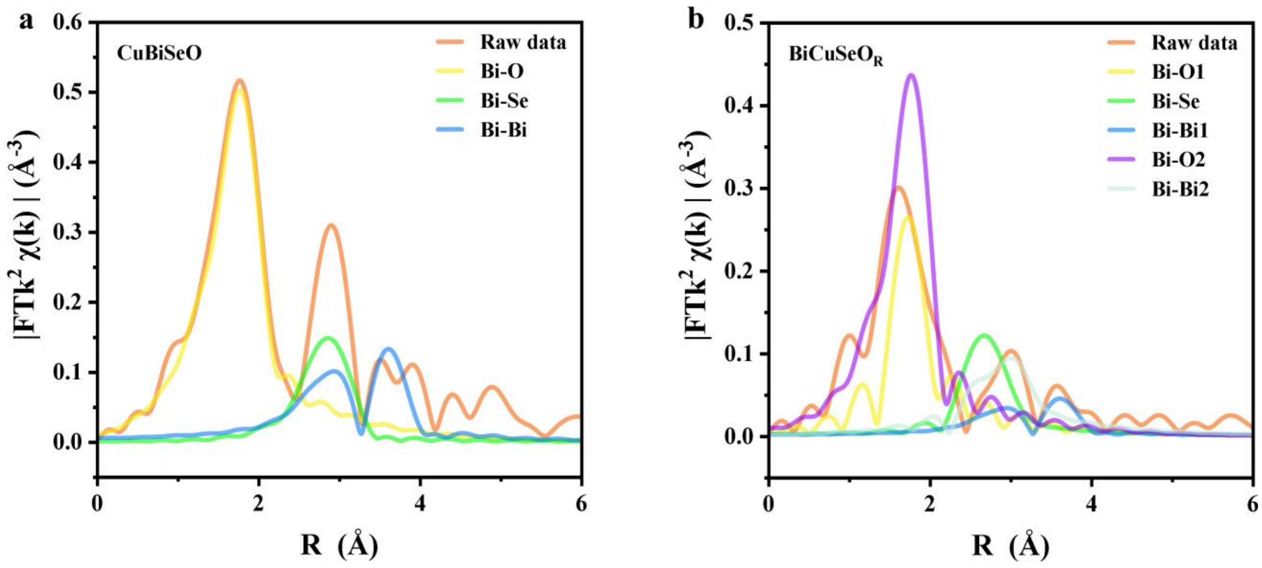

Supplementary Fig. S18 Bi L3-edge EXAFS fitting curves of (a) BiCuSeO and (b) $BiCuSeO_R$ samples. By fitting Bi L3-edge EXAFS of $BiCuSeO_R$, the first nearest backscattering path of Bi-O includes two coordination states of Bi-O1 and Bi-O2.

S19. Structural characterization of Bi_2O_3 before and after CO_2RR

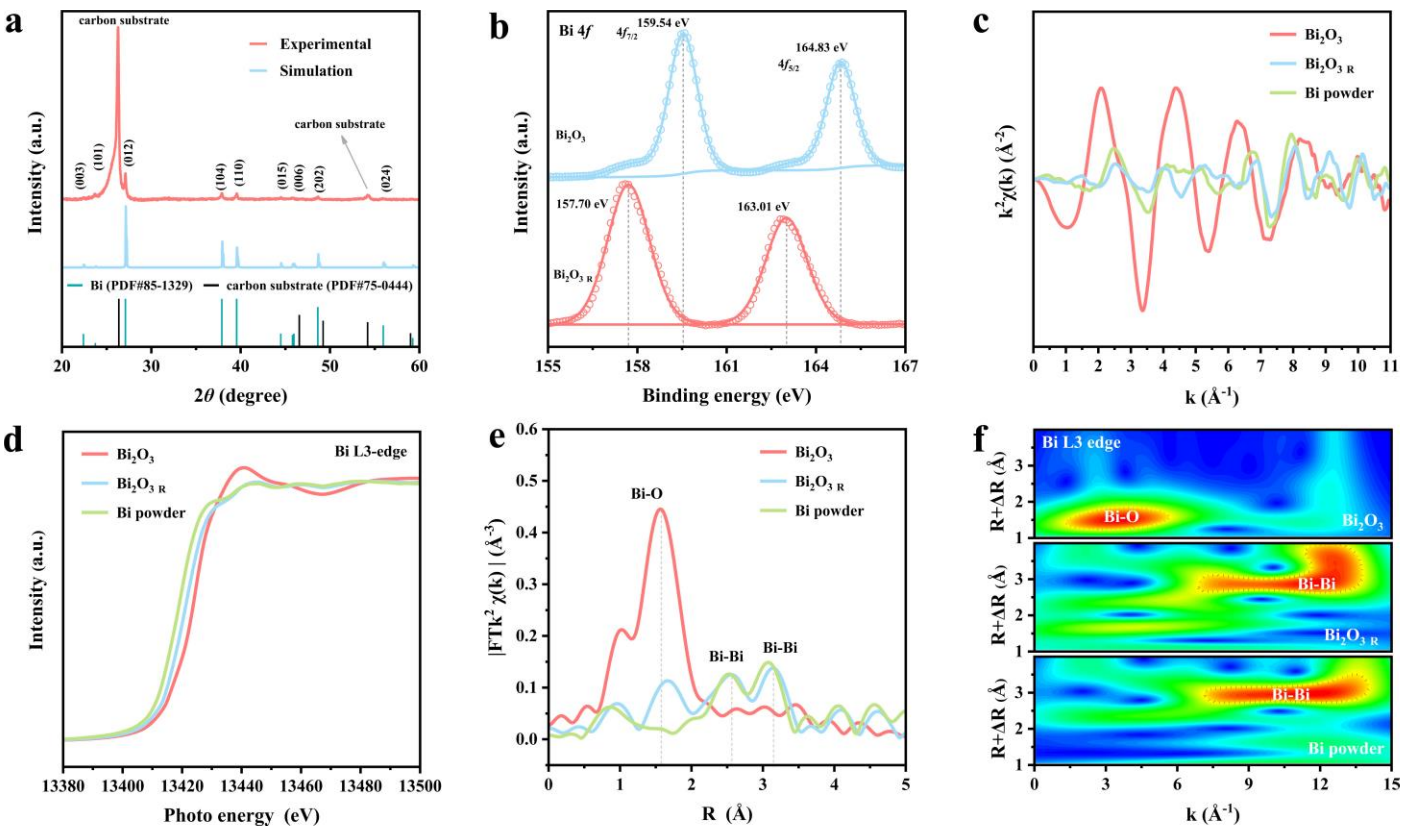

Supplementary Fig. S19 (a) XRD pattern. (b) Bi 4*f* XPS spectra. (c) Bi L3-edge $k^2\chi(k)$ oscillation functions curves. (d) XANES spectra. (e) EXAFS spectra. (f) Wavelet transform of the k^2-weighted EXAFS data (WT-EXAFS). Bi_2O_3 before and after CO_2RR are named as Bi_2O_3 and $Bi_2O_{3\ R}$ respectively. Besides the peaks of Carbon substrate for catalysts depositing, all the peaks in the XRD pattern can correspond to Bi (PDF# 85-1329), suggesting that Bi_2O_3 is reduced to metallic Bi after CO_2RR. Peaks corresponding to zero valent metal in Bi 4*f* XPS spectra further confirm the easy self-reduction of Bi_2O_3 during electrocatalytic CO_2 reduction reaction. Bi L3-edge EXAFS $k^2\chi(k)$ oscillation functions curves, XANES spectra, EXAFS spectra and WT-EXAFS consistently display that Bi_2O_3 are mostly reduced to the metal bismuth after CO_2RR.

S20. Structural characterizations of CuSe-BiO samples collected after CO_2RR

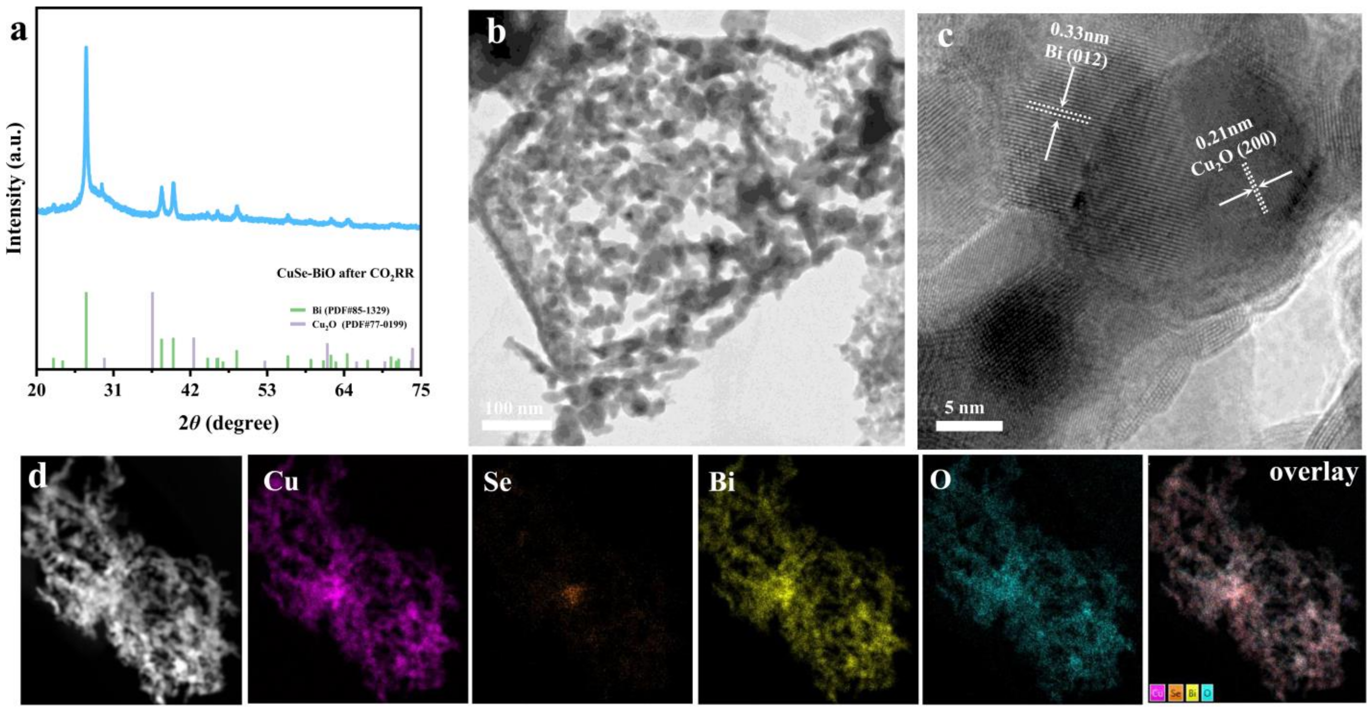

Supplementary Fig. S20 Structural characterizations of Cu_2Se-Bi_2O_3 hetero-structures after the CO_2RR. (a) XRD pattern. (b) TEM image. (c) HRTEM image. (d) EDS mapping images. Bi and Cu_2O phase appear in the XRD pattern, suggesting that Bi_2O_3 is reduced to metallic Bi, while Cu_2Se is transformed into Cu_2O for CuSe-BiO after the CO_2RR. TEM image shows that CuSe-BiO has been transformed into polycrystalline nanoparticles. The lattice spacing of 0.21 and 0.33 nm corresponding to Cu_2O (200) plane and Bi (012) plane respectively in HRTEM image confirm the generation of Bi and Cu_2O after the CO_2RR. In addition, unform Cu, Bi, O distribution and the indistinct Se distribution in EDS mapping explains the massive loss of Se (Supplementary Fig. S20d).

S21 Cu K-edge EXAFS Fitting

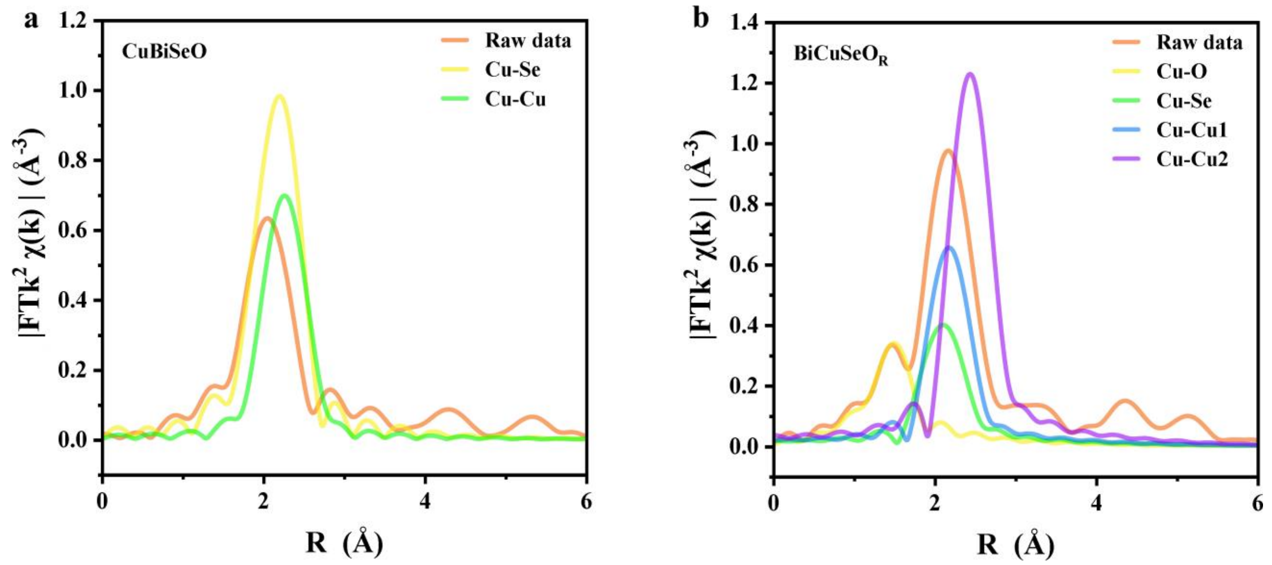

Supplementary Fig. S21 Cu K-edge EXAFS fitting cures of (a) BiCuSeO and (b) $BiCuSeO_R$ samples. By fitting Cu K-edge EXAFS of $BiCuSeO_R$, a new peak appearing at ~1.5 Å can be corresponded to Cu-O. Moreover, the sharply reducing peak intensity of Cu-Se evidentially illustrates that Se atoms escape from BiCuSeO during CO_2RR.

S22. In situ Cu K-edge XAFS spectra of BiCuSeO at different potentials

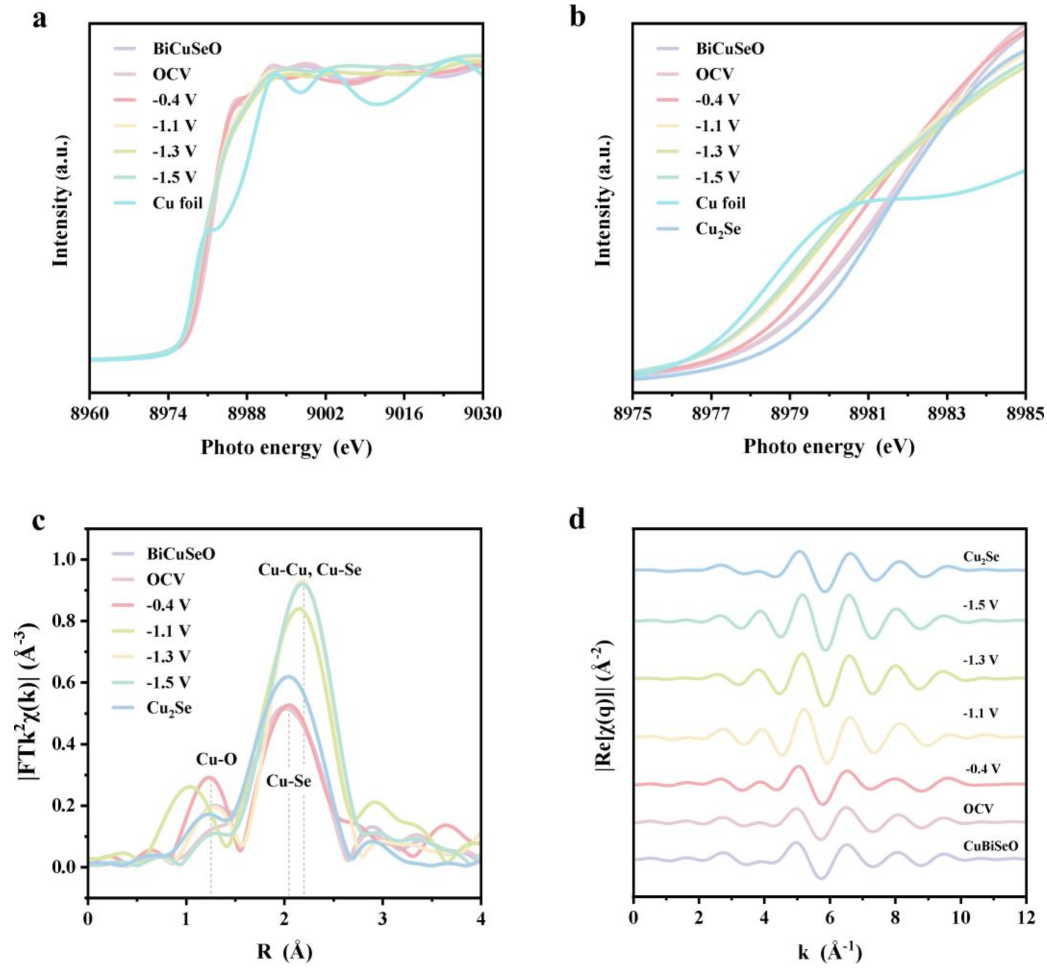

Supplementary Fig. S22 (a, b) Cu K-edge XANES spectra of BiCuSeO at different potentials and Cu foils. (c) Corresponding Fourier transform of Cu K-edge EXAFS spectra of BiCuSeO at different potentials and Cu foils. (d) Fitted k-space EXAFS spectra of BiCuSeO at different potentials and Cu foils reference. In order to monitor the structural changes of BiCuSeO during the CO_2RR, in situ XAFS measurement is conducted. From the Cu K-edge XANES spectra, the absorption edge and white line peak of BiCuSeO under OCV aligns well with pristine BiCuSeO and Cu_2Se sample, suggesting its monovalent feature. As the potential is decreased from -0.4 V to -1.3 V, the absorption edge shifts to lower energy gradually and keeps unchanged at -1.5 V. This combined with the EXAFS spectra indicate the massive escape of Se atoms. A new peak at ~1.29 Å corresponding to the Cu-O bond occurs in EXAFS spectra suggests O entrance. It is worth noting that there remain peak features in the R=1~4 Å range under all operating potentials, BiCuSeO structure can be maintained to a great degree. Taken together, the above results illustrate Cu-Se bonds breaking, Se atoms escaping, concomitant O entrance and Cu aggregation take place in BiCuSeO under CO_2RR.

S23. Cu XPS spectra of BiCuSeO before and after CO_2RR

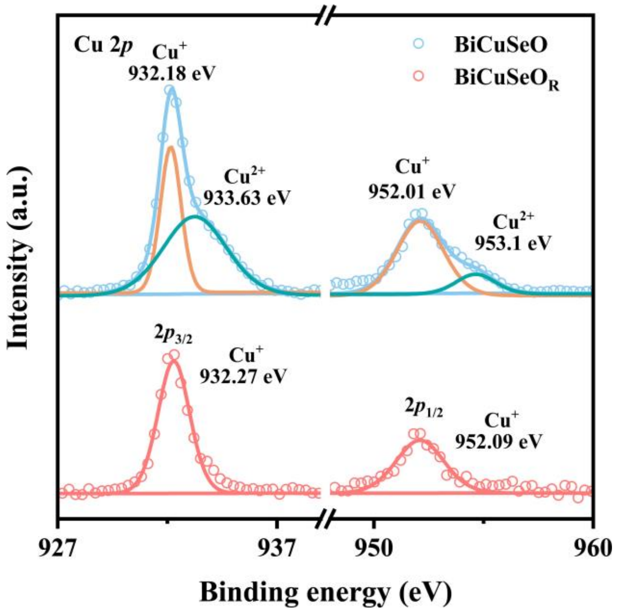

Supplementary Fig. S23 Cu XPS spectra of BiCuSeO catalysts before and after CO_2RR.

S24. The structural characterization of Cu_2Se before and after CO_2RR

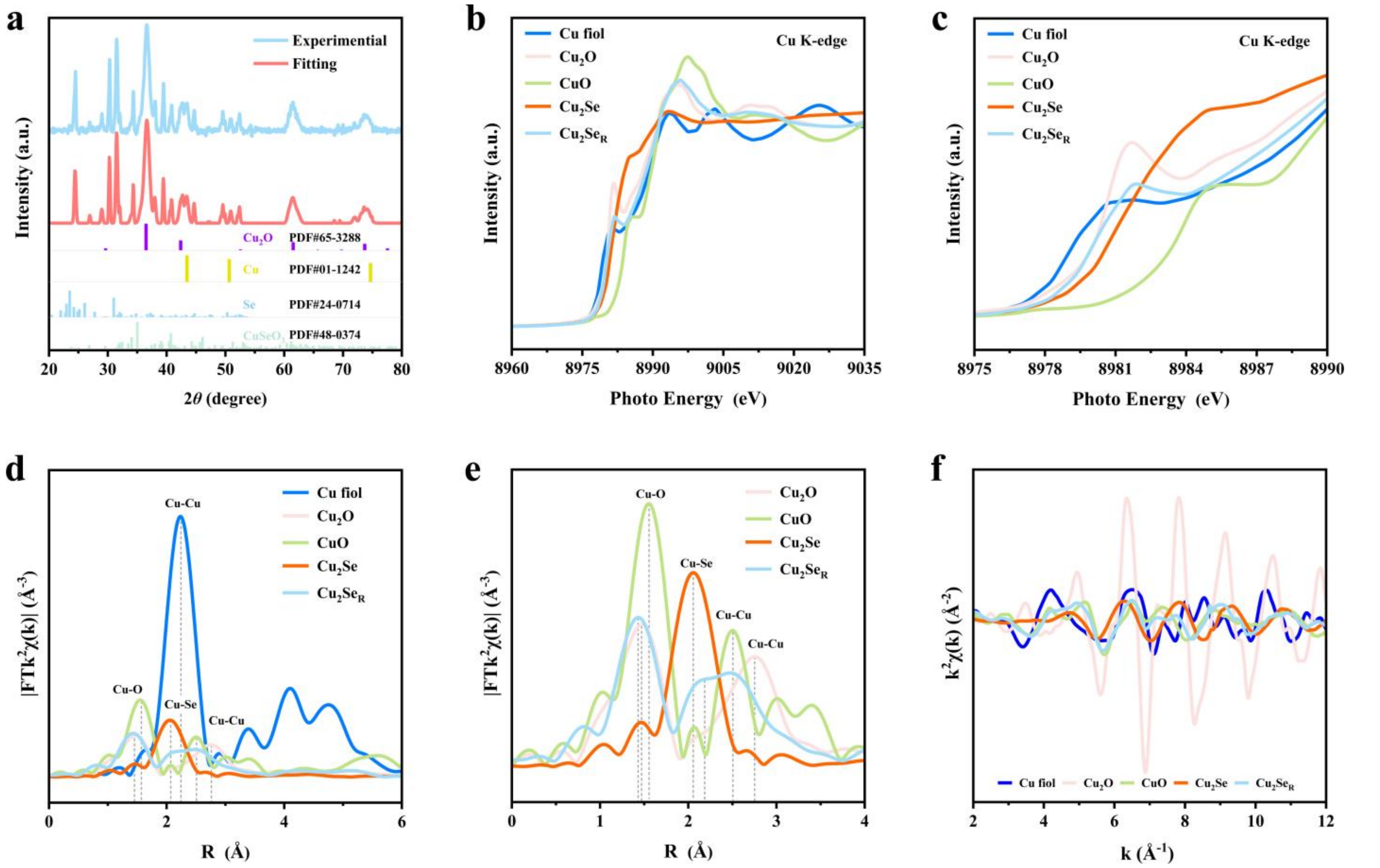

Supplementary Fig. S24 (a) Experimental and fitting XRD patterns. (b, c) Cu K-edge XANES of Cu_2Se, Cu_2Se_R and reference samples. (d, e) FT-EXAFS spectra of Cu_2Se, Cu_2Se_R and reference samples. (f) Cu K-edge $k^2\chi(k)$ oscillation functions curves of Cu_2Se, Cu_2Se_R and reference samples. XRD pattern shows that Cu_2Se is dominantly transformed into Cu_2O and Cu, complying a small quality of $CuSeO_3$. XANES and EXAFS spectra also demonstrates that valence states of Cu atoms in Cu_2Se_R include the mixture of monovalent and divalent Cu, which is consistent with the XRD results.

S25. XPS spectra of Cu_2Se before and after CO_2RR

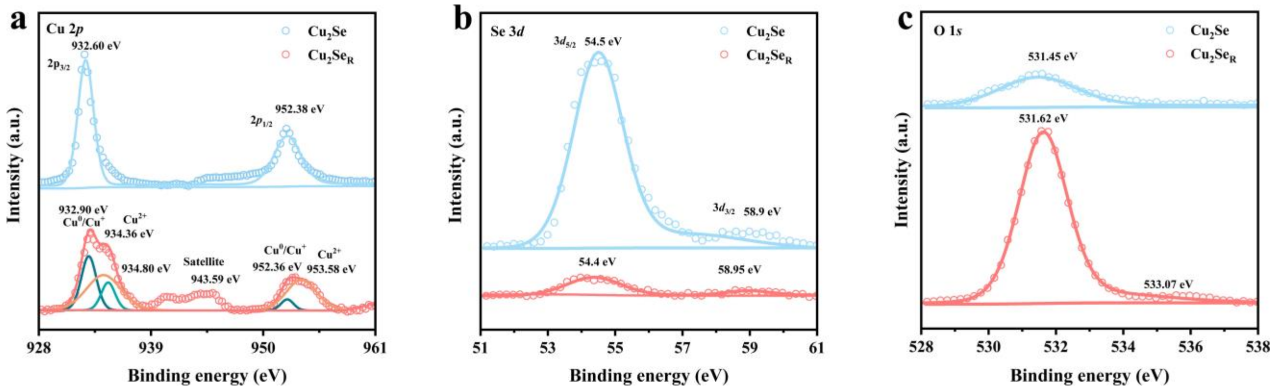

Supplementary Fig. S25 XPS spectra of Cu_2Se before and after CO_2RR. (a) Cu 2*p*, (b) Se 3*d*, and (c) O 1*s* XPS spectra of Cu_2Se after CO_2RR. After CO_2RR, typical peaks of Cu^0, Cu^+ and Cu^{2+} can be observed in the Cu 2*p* XPS spectra, indicating partial Cu is reduced and oxidized. Moreover, the peak of the Se $3d_{5/2}$ for Cu_2Se after CO_2RR shows a sharp decrease, suggesting the massive escaping of Se atoms. Meanwhile, greatly enhanced peak intensity of O 1*s* reveals that the entrance of O. XPS results are in well agreed with the XRD, XANES and EXAFS analyses.

S26. Structural model of pourbaix diagram

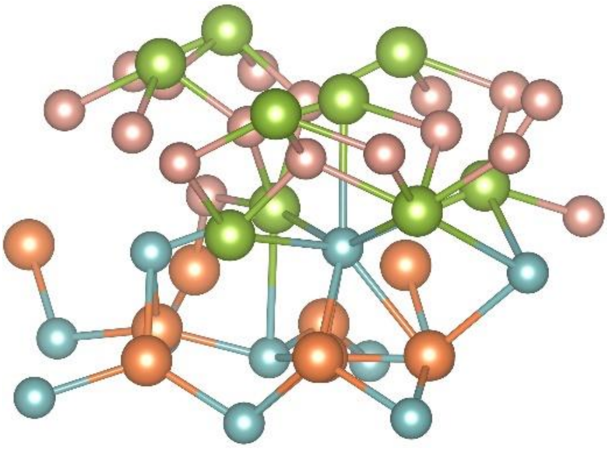

Supplementary Fig. S26 Calculation model of oxygen defects for pourbaix diagram.

S27. Structural models of charge density, charge density difference and DOS

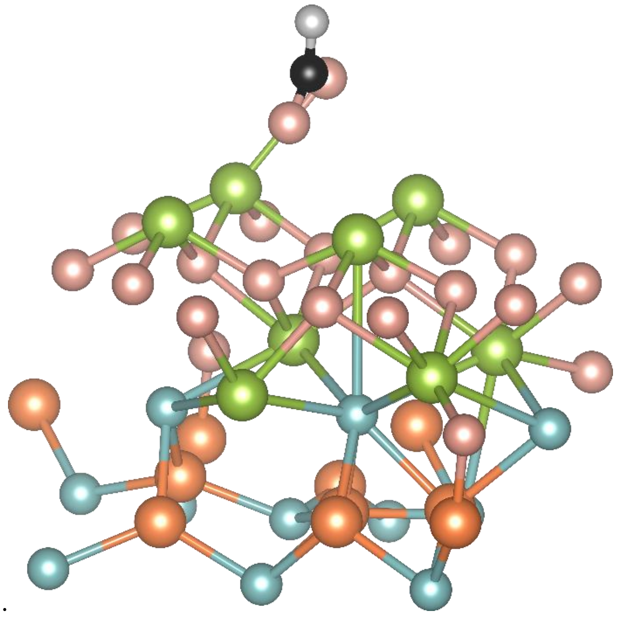

Supplementary Fig. S27 Structural models of charge density, charge density difference and DOS.

S28. Inter-sublayer charge separation

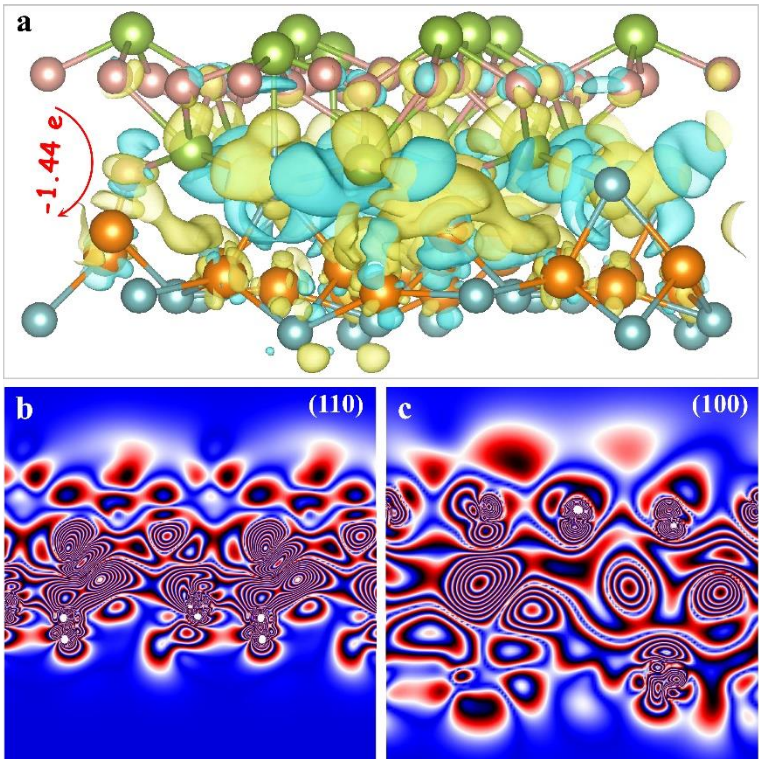

Supplementary Fig. S28 Inter-sublayer charge separation. The calculation result reveals that $[Cu_2Se_2]^{2-}$ sublayer can efficiently capture electrons, while the $[Bi_2O_2]^{2+}$ sublayer are depleted of electrons. And the accumulated sublayer charge quantity is -1.44 e. The above results suggest that the conductive $[Cu_2Se_2]^{2-}$ sublayer efficiently conducts electrons to protect the active center of the $[Bi_2O_2]^{2+}$ sublayer to drive the activation of CO_2 molecules during the electrochemical CO_2RR.

S29. O p, Bi p, Bi s and Cu p orbitals PDOS of BiCuSeO during CO_2RR

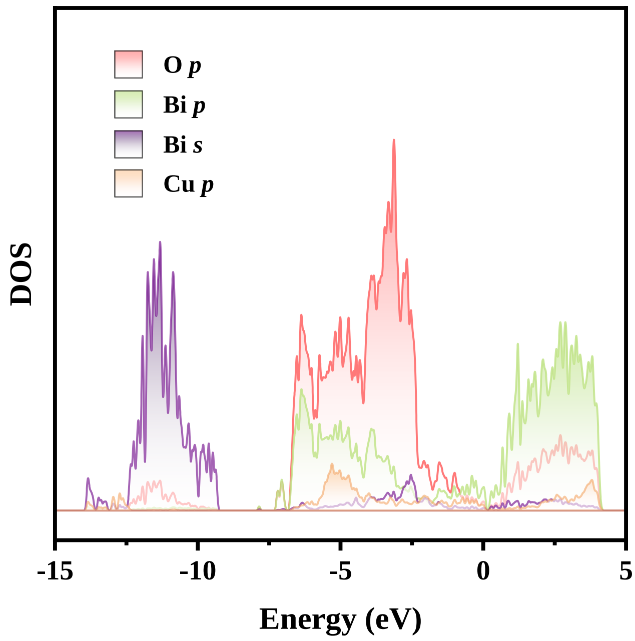

Supplementary Fig. S29 O *p*, Bi *p*, Bi *s* and Cu *p* orbitals PDOS of BiCuSeO during CO_2RR. PDOS of Bi *p* and Bi *s* orbitals overlap with that of O *p* orbitals to a great extent, indicating a strong interaction between Bi atoms and O atoms, and the oxide state of Bi in $[Bi_2O_2]^{2+}$ sublayer is negligibly influenced by structural transformation and can be well retained.

S30. Total DOS

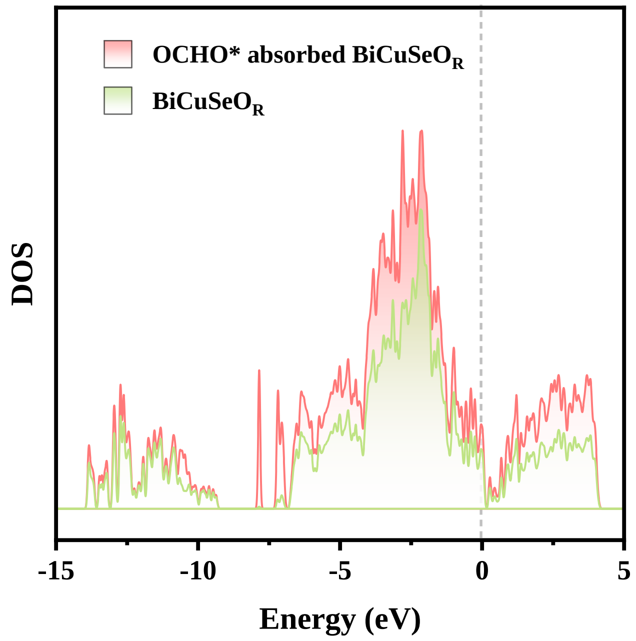

Supplementary Fig. S30 Total DOS comparisons of BiCuSeO$_R$ and OCHO* absorbed on BiCuSeO$_R$.

S31. Structural models of intermediates for IS, TS and FS

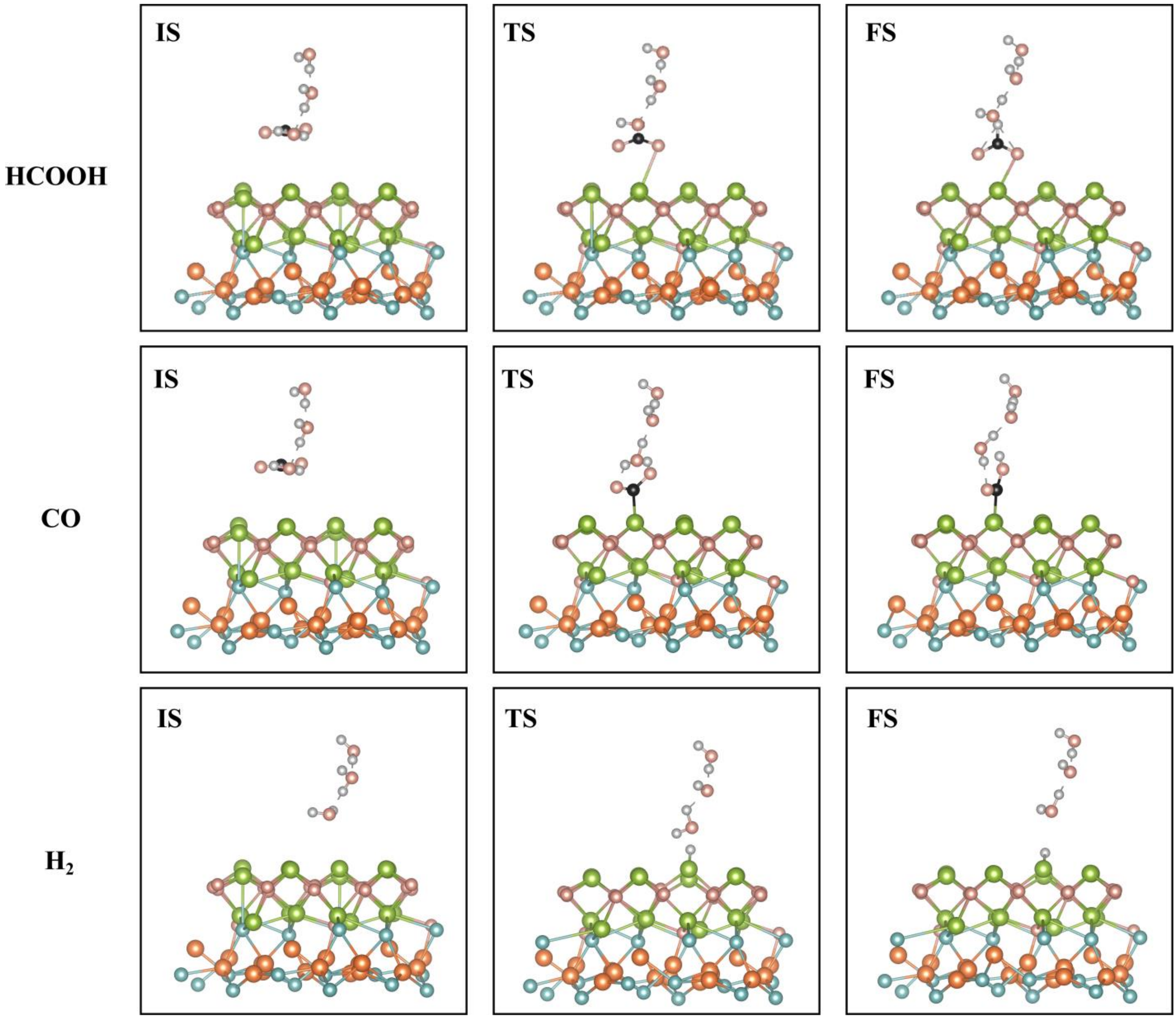

Supplementary Fig. S31 Structural models of intermediates for IS, TS and FS.

S32. Structural model of OCHO* intermediate

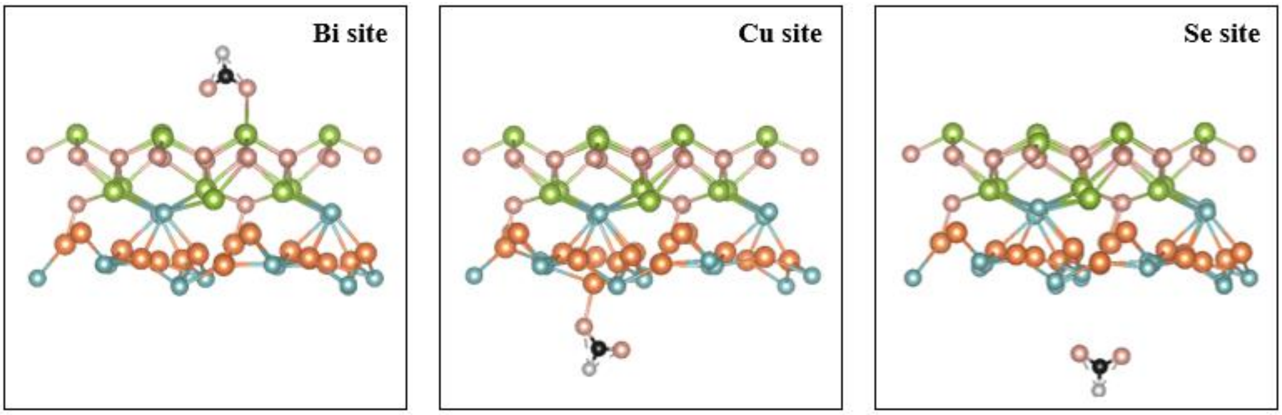

Supplementary Fig. S32 Structural model of OCHO* intermediate absorbed on Bi, Cu and Se sites.

S33. Calculated hydrogen absorbed on $BiCuSeO_R$ model

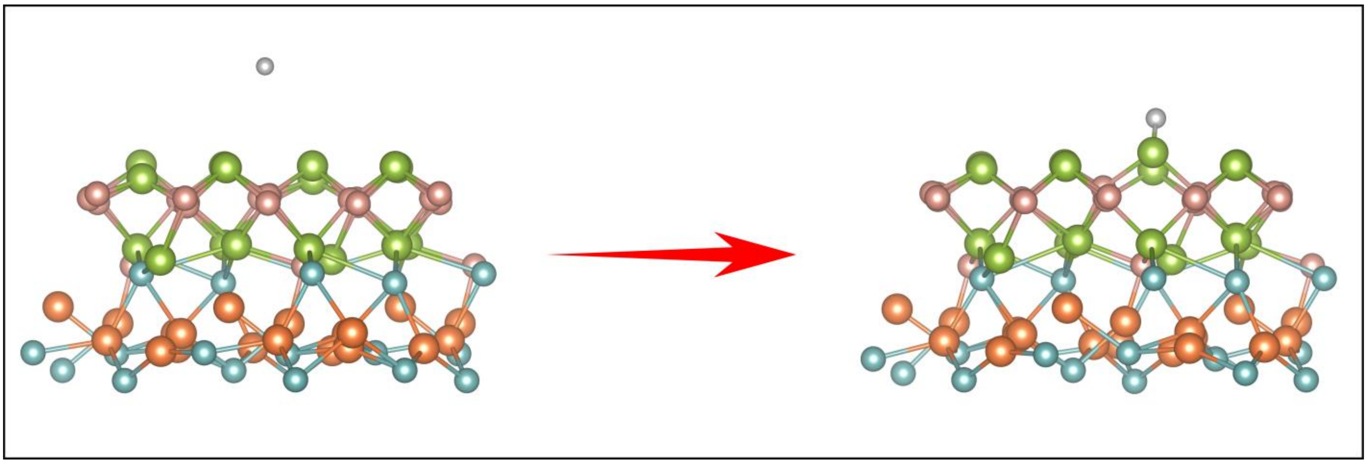

Supplementary Fig. S33 Calculated hydrogen absorbed on $BiCuSeO_R$ model. According to structural model optimization by theoretical calculation, hydrogen tends to be adsorbed at the Bi site, and the free energy formation of HER calculated by using the corresponding structural model.

S34. Reaction pathways of formate (HCOOH) producing and leaving

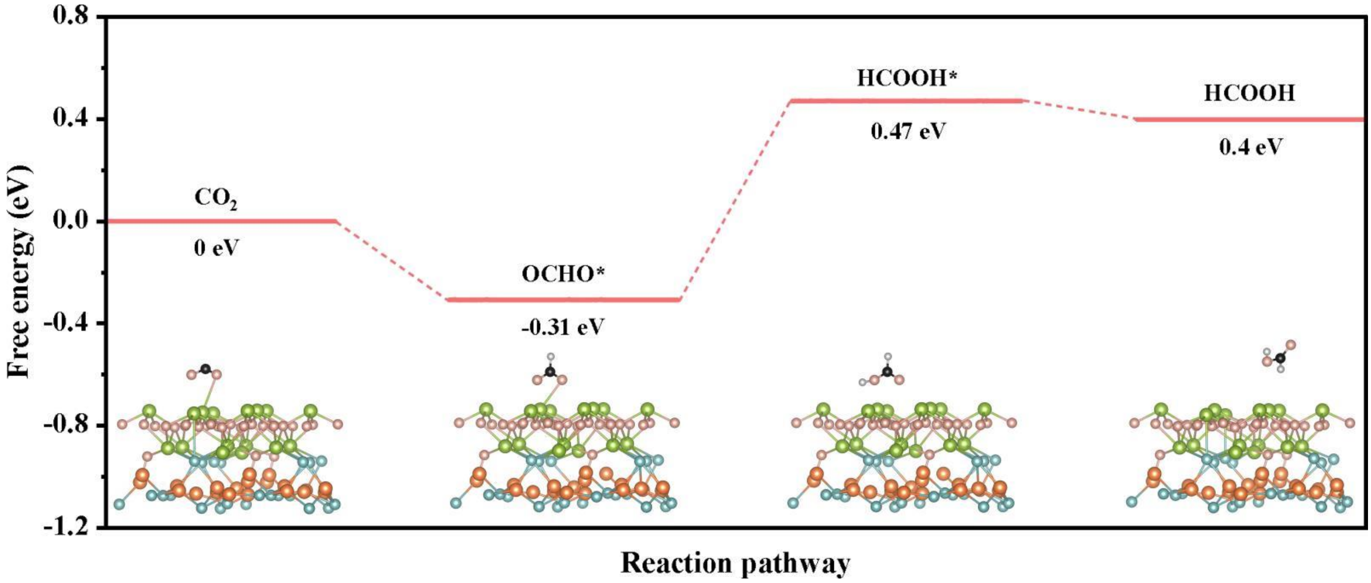

Supplementary Fig. S34 Proposed reaction pathway of formate producing and leaving. Firstly, CO_2 molecule is bonded and activated on Bi site of BiCuSeO, and then forms the intermediate species $*CO_2^{\bullet -}$ with the assistant of one electron transfer ($CO_2 + e^- = *CO_2^-$). Subsequently, the intermediate species OCHO* will be formed by coupling a proton ($*CO_2^- + H^+ =$ OCHO*). After further one electron and proton transfer, OCHO*can be reduced into formate (OCHO* + H^++ e^- = HCOOH), and finally desorbs from catalytic site surface. Notably, DFT calculation shows that it is a physical adsorption for formate absorbing on the positive-charged $[Bi_2O_2]^{2+}$ layer. And the calculated Gibbs free energy for the desorption of formate from $[Bi_2O_2]^{2+}$ layer is -0.07 eV, indicating formate desorption processes is spontaneous.

S35. Schematic illustration of in situ XAFS device

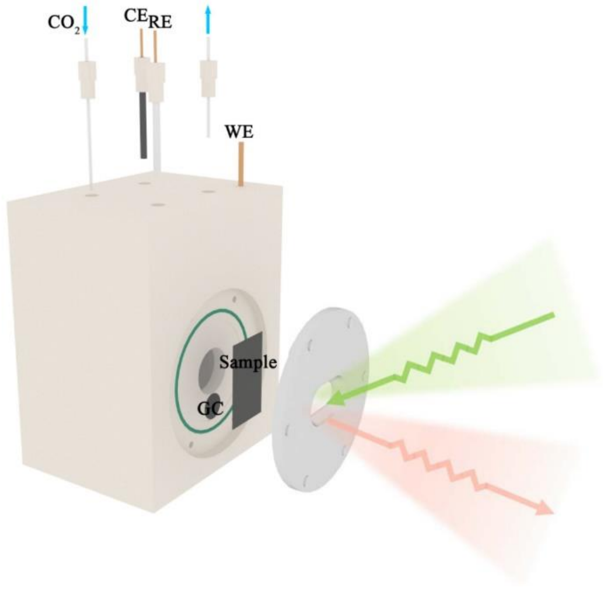

Supplementary Fig. S35 Schematic illustration of in situ XAFS device. The working electrode (WE) is a catalyst-deposited on carbon paper (Sample), and connected by a glassy carbon electrode (GC). The reference electrode (RE) and counter electrode (CE) are Ag/AgCl and carbon rod, respectively. CO_2 gas is supplied before in situ XAFS test. Electrolyte is 0.5 M $KHCO_3$ solution.

Supplementary Table S1 CO_2 RR product distributions for BiCuSeO, Cu_2Se, Bi_2O_3 and CuSe-BiO.

***E* (V)**	**$FE_{formate}$** (%)				**FE_{CO}** (%)				**FE_{H2}** (%)			
	BiCuSeO	Cu_2Se	Bi_2O_3	CuSe-BiO	BiCuSeO	Cu_2Se	Bi_2O_3	CuSe-BiO	BiCuSeO	Cu_2Se	Bi_2O_3	CuSe-BiO
-0.4	93.74	46.5	81.5	52.1	0	0.32	0	0.3	1.45	18.11	5.51	47.5
-0.5	93.42	57	80.7	72.3	0.02	0.28	0.06	0.3	6.56	33.91	12.81	18.4
-0.6	90.25	52.1	83.5	62.4	0.11	0.14	0.23	0.6	9.64	37.13	11.77	35.5
-0.7	90.29	50.7	84.6	69.1	6.21	0.43	0.62	2.2	3.5	48.07	16.3	27.8
-0.8	92.9	61.05	86.2	81.4	2.4	0.87	2.55	2	4.7	28.98	13.05	7.3
-0.9	93.4	54.92	84.8	89.8	2.36	0.51	3.21	2.9	3.35	41.42	12.91	8.3
-1.0	90.02	53.27	82.3	71.4	3.87	2.52	2.26	2.9	6.11	43.61	13.02	15.5
-1.1	90.24	44.08	81.4	52.6	2.75	6.24	3.33	2.1	7.01	44.57	15.55	28.7

Supplementary Table S2. CO_2RR comparison of Bi-based electrocatalysts for formate production.

	Samples	FE_{max}/E(V)	FE_{min}/E(V)	E_{max}(V)	E_{min} (V)	$FE_{[E_{min}]}$	Overpotential (mV)	iR (Y/N/U)	References
1	β-Bi_2O_3fractals	87%/-1.2	27%/-0.8	-1.2	-0.8	27%	590	N	Adv. Funct. Mater. 2020, 30, 1906478
2	Bi Ns	86%/-1.1	33%/-0.5	-1.2	-0.5	33%	290	N	Nano Energy 2018, 53, 808-816
3	Bi_2O_3 sphere	90%/-0.9	62%/-0.7	-1.1	-0.7	62%	490	U	ACS Catal. 2020, 10, 1, 743-750
4	Bi_2O_3NPs@C	93%/-0.9	80%/-0.7	-1.1	-0.7	80%	490	U	Angew. Chem. Int. Ed. 2020, 59, 10807-10813
5	$BiPO_4$NSs	92%/-0.9	74%/-0.8	-1.2	-0.8	74%	590	U	Angew. Chem. Int. Ed. 2021, 60, 7681-7685
6	Bi_2O_3Ns/CNT	93.6%/-1.256	14%/-0.556	-1.356	-0.556	14%	346	N	Angew. Chem. Int. Ed. 2019, 58, 13828-13833
7	Bi NSs	100%/-0.7	3%/-1.1	-1.1	-0.7	100%	490	Y	Angew. Chem. Int. Ed. 2020, 59, 20112-20119
8	Bi NTs	97%/-0.85	5%/-0.38	-1.05	-0.38	5%	170	N	Nat. Commun. 2019, 10, 2807
9	Bi NSs	95%/-0.9	18%/-0.603	-1.06	-0.51	<1%	300	N	Nat. Commun. 2018, 9, 1320
10	Bi dendrites	~89%/-0.74	29%/-1.18	-1.18	-0.56	64%	350	U	ACS Catal. 2017, 7, 8, 5071-5077
11	Bi NWs	95%/-0.7	41%/-0.5	-1.19	-0.5	41%	290	Y	Energy Environ. Sci., 2019, 12, 1334-1340
12	Bi nanoflakes	100%/-0.9	79.5%/-0.4	-1.2	-0.4	79.50%	190	N	Nano Energy 2017, 39, 44-52
13	BiO_x/C	97%/-0.92	22%/-0.65	-1.4	-0.65	22%	440	Y	ACS Catal. 2018, 8, 2, 931-937
14	Bi NSs	99%/-0.9	56%/0.75	-0.95	-0.75	56%	540	Y	Adv. Mater. 2018, 30, 1802858
15	Bi-Sn/CF	96%/-1.14	44%/-0.64	-1.24	-0.64	44%	420	U	Adv. Energy Mater. 2018, 8, 1802427
16	**BiCuSeO**	93.4%/-0.9	90.02%/-1.0	-1.1	-0.4	~93%	190	N	**This work ★**

Note: Y: with iR compensation; N: without iR compensation; U: undefined.

Supplementary Table S3. ECSA calculation results.

Samples	Cdl (mF cm^{-2})	ECSA
BiCuSeO	5.66	94.3
Cu_2Se	5.59	93.2
Bi_2O_3	4.91	81.8
CuSe-BiO	4.48	74.7

Supplementary Table S4 Computed vibrational features for the intermediate *CO_2.

Raman shift (cm^{-1})	593.81	593.81	1295.1	2458.98
Vibrational feature	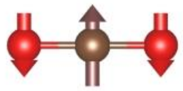	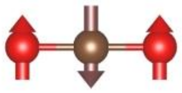	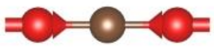	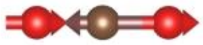

Supplementary Table S5 Computed vibrational features for the intermediate OCHO*.

Raman shift (cm^{-1})	990.23	1252.92	1324.14	1544.98	2458.98
Vibrational feature	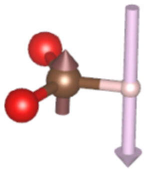	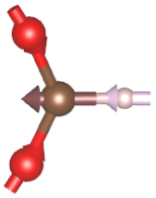	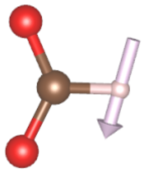	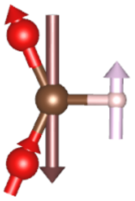	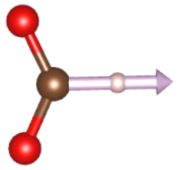

Supplementary Table S6. EXAFS curves fitting results.

XAS edge	Sample	Shell	N	R (Å)	σ^2*10^3 (Å^2)	R-factor (%)
Bi L3-edge	Bi_2O_3	Bi-Bi	3	3.06	/	/
		Bi-O	3	2.13	/	/
	BiCuSeO	Bi-O	4	2.29	8.9	1.2
		Bi-Se	8	3.25	15.0	
		Bi-Bi	4	3.72	15.3	
	$BiCuSeO_R$	Bi-O1	0.4	2.13	6.1	0.6
		Bi-O2	2.5	2.27	4.5	
		Bi-Bi1	0.3	3.09	3.3	
		Bi-Se	0.8	3.15	5.2	
		Bi-Bi2	2.4	3.71	15.1	
Cu K-edge	Cu foil	Cu-Cu	12	2.56	/	/
	BiCuSeO	Cu-Se	4	2.47	11.6	0.8
		Cu-Cu	4	2.72	9.4	
	$BiCuSeO_R$	Cu-O	0.8	1.93	9.9	0.3
		Cu-Se	0.7	2.43	3.6	
		Cu-Cu	4.0	2.58	12.5	

N, coordination number; R, distance between absorber and backscatter atoms; σ^2, Debye-Waller factor to account for both thermal and structural disorders; R-factor (%) indicate the goodness of the fit. The obtained S_0^2 of Cu foil was 0.87 and it was fixed in the subsequent fitting of Cu foil K-edge data for the catalyst.The obtained S_0^2 of Bi_2O_3 standard was 0.79 and it was fixed in the subsequent fitting of Bi foil L3-edge data for the catalyst.

Supplementary Table S7. The vibrational frequencies of the reaction intermediates.

*COOH	f (cm^{-1})	OCHO*	f (cm^{-1})	H*	f (cm^{-1})
1f	3479.10	1f	2884.84	1f	1193.50
2f	1542.97	2f	1516.68	2f	571.49
3f	1229.46	3f	1326.90	3f	324.44
4f	1007.19	4f	1310.12		
5f	690.39	5f	1014.67		
6f	628.46	6f	746.23		
7f	287.87	7f	231.92		
8f	226.92	8f	189.60		
9f	200.60	9f	181.63		
10f	140.48	10f	169.24		
11f	99.46	11f	85.49		
12f	80.71	12f	58.39		

Supplementary Table S8. Zero-point energy, enthalpic correction and entropy correction at 298.15 K.

**Species**	$\boldsymbol{E_{ZPE}}$ **(eV)**	**TS (eV)**	$\boldsymbol{G_{corr}}$ **(eV)**
*COOH	0.6	0.21	0.39
OCHO*	0.6	0.23	0.37
H*	0.13	0.02	0.11

Supplementary Note 1. Possible structural transformation of BiCuSeO during CO_2RR.

From the XAFS, TEM, and XPS analysis results, $BiCuSeO_R$ also maintain the crystalline phase, morphologies and structure of tetragonal BiCuSeO. The construction process of $BiCuSeO_R$ is simply proposed in Fig. 5a. During the electrocatalytic CO_2RR process, the conductive Cu_2Se_2 layer are firstly interplayed by the negative potential and the Se atoms instantly escaped from the Cu_2Se_2 sublayers. With breaking of Cu-Se bonds, partial Se atoms are replaced in situ by the strong electronegative O to form Cu-O bonds, at the same time Cu-Cu bonds are generated locally. Due to the confinement of Bi_2O_2 layer, the frame of Cu_2Se_2 layer tend to be stable. Meanwhile, the Bi_2O_2 sublayer are protected and mainly kept because the conductive Cu_2Se_2 sublayer rapidly conducts electrons.

Supplementary Note 2. Detailed Raman calculation.

The plane wave package Quantum Espresso[9] was used to perform density functional theory calculations. Generalized gradient approximation (GGA) functional[10] and norm-conserving SG15 pseudopotential[11] were used for the structural relaxation and the computation of vibrational properties. The plane wave energy cutoff of 50 Ryd is found to yield converged charge density. The forces and phonons are computed with a 2×2×1 k-point grid sampling. Here, the vacuum between two slabs is set to be at least 15 Å to avoid image-image interactions. All the structures are fully relaxed with forces below 0.0025 eV/Å. For the relaxed structure, a zone-center phonon calculation was performed with density functional perturbation method (DFPT)[12] implemented in Quantum Espresso. The acoustic sum rule was applied after the phonon calculation to correct the phonon eigen modes and eigen energies.

Supplementary Note 3. Detailed DFT calculation.

The formation energy of $[Bi_2O_2]^{2+}$ is defined as:

$$E_{\mathrm{form}} = E_{\mathrm{Bi2O2}} - N_{\mathrm{Bi}}\mu_{\mathrm{Bi}} - N_{\mathrm{O}}\mu_{\mathrm{O}} \quad \text{(S1)}$$

Where E_{Bi2O2} is the total energy of bulk Bi_2O_2, and N_{Bi}, and N_{O} are the number of Bi and O, respectively. The term μ denotes the chemical potential of species, μ_{Bi} and μ_{O} are taken as atom energy of bulk Bi (ΔE_{hull}=0) and O_2 molecule. According our calculations, the formation energy of Bi_2O_2 is -1.64 eV, which demonstrate that this structure can maintain stability, experimentally.

Under the CO_2 reduction conditions (liquid water at T = 298.15 K), which can be expected to cause an accumulation of H atoms on the metal oxide sublayer surface, it may result in oxygen reduction and then it could be form the oxygen defects. The relevant reaction and free energy change are shown below.

$$\mathrm{CuSeBiO_n} + \mathrm{H^+} + \mathrm{e^-} \rightarrow \mathrm{O_{vac}\text{-}CuSeBiO_{n-1}} + \mathrm{H_2O} \quad \text{(S2)}$$

$$\Delta G = G(H_2O) + G(O_{vac}\text{-CuSeBiO}_{n-1}) - G(\text{CuSeBiO}_n) - 0.5G(H_2) + eU_{SHE} + k_B T\ln(10)pH \quad (S3)$$

where the $G(O_{vac}\text{-CuSeBiO}_{n-1})$ and $G(\text{CuSeBiO}_n)$ are the total energy of entire CuSeBiO and CuSeBiO with an oxygen defect, respectively.

The adsorption energy of HCOOH on CuSeBiO monolayer was calculated by:

$$\Delta G_{ad} = G_{tot} - G_{CuSeBiO*} - G_{HCOOH} \quad (S4)$$

where G_{tot}, $G_{CuSeBiO*}$, and G_{HCOOH} are the DFT energy of HCOOH absorbed on CuSeBiO monolayer, adsorbate-free CuSeBiO monolayer, and HCOOH molecule, respectively.

The Gibbs free energy of electrochemical reactions were calculated using the computational hydrogen electrode (CHE), and All the calculations were carried out based on density functional theory (DFT) as implemented in the Vienna Ab initio Software Package code within the Perdew-Burke-Ernzerhof (PBE) generalized gradient approximation (GGA) and the projected augmented wave (PAW) method.[13-16] The cutoff energy of the plane-wave basis is 500 eV, and the Brillouin zone was sampled with 3 × 3 × 1 and 4× 4× 1 k-points for geometry optimization and electronic structure computations, respectively. The convergence criterion for the electronic self-consistent iteration and force was set to 10^{-5} eV and 0.01 eV/Å, respectively. The distance of vacuum space was set to about 30.0 Å. The van der Waals (vdW) interactions were included using Grimme's D3 method.[17] Poisson-Boltzmann implicit solvation model was used to consider solvation effect and the cavity setting in VASPsol was turned off to avoid numerical instabilities.[18,19] The climbing-image nudged elastic band (CI-NEB) method was employed to simulate the reaction energy barrier.[20]

The Gibbs free energy of electrochemical reactions were calculated using the computational hydrogen electrode (CHE),[21] and it could be computed by:

$$\Delta G = \Delta E + \Delta E_{ZPE} - \Delta TS + \Delta G_{pH} + \Delta G_U \quad (S5)$$

where E is the ground state energy, E_{ZPE} and S (T = 298 K) are the zero-point energy difference and the entropy, respectively. For each system, E_{zpe} can be calculated by summing vibrational frequencies over all normal modes ν (E_{zpe} = 1/2Σhν). The effects of pH and electrode potential (U) can be treated as: ΔG_{pH}=0.0592×pH and ΔG_U=-eU. To obtain the proper absolute electrochemical, the work function (Φ) of material should be adjusted by adding electrons.[22,23] It could be calculated by:

$$U = (W_f - 4.44) + 0.0592\times pH \quad (S6)$$

where Φ is the work function relative to reference level, (4.40-0.0592×7) eV is introduced to account for the work function of reversible hydrogen electrode (RHE). According to Neurock methods,[24] the potential-dependent energy can be calculated by:

$$E_{free}(U) = E_{DFT} + \int_0^q \langle \overline{V_{tot}} \rangle dQ + qW_f \quad (S7)$$

Supplementary References

1. Zhao, L. D. *et al.* BiCuSeO oxyselenides: new promising thermoelectric materials. *Energy Environ. Sci.* **7**, 2900-2924 (2014).
2. Samanta, M., Guin, S. N. & Biswas, K. Ultrathin few layer oxychalcogenide BiCuSeO nanosheets. *Inorg. Chem. Front.* **4**, 84-90 (2017).
3. Zhu, H. *et al.* Efficient interlayer charge release for high-performance layered thermoelectrics. *Natl. Sci. Rev.* **8**, nwaa085 (2021).
4. Viennois, R. *et al.* Lattice dynamics study of thermoelectric oxychalcogenide BiCuChO (Ch = Se, S). *J. Phys. Chem. C* **123**, 16046-16057 (2019).
5. Yang, B., Yang, J., Huang, Z., Qin, L., Lin, H. & Li, Q. Green fabrication of large-size Cu_2Se hexagonal sheets with visible light photocatalytic activity. *Appl. Surf. Sci.* **535**, 147712 (2021).
6. Bohra, D., Ledezma-Yanez, I., Li, G., de Jong, W., Pidko, E. A. & Smith WA. Lateral adsorbate interactions inhibit HCOO- while promoting CO selectivity for CO_2 electrocatalysis on silver. *Angew. Chem. Int. Ed.* **58**, 1345-1349 (2019).
7. Chernyshova, I. V., Somasundaran, P. & Ponnurangam, S. On the origin of the elusive first intermediate of CO_2 electroreduction. *Proc. Natl Acad. Sci. USA* **115**, E9261-E9270 (2018).
8. Stanila, D., Smith, W. & Anderson, A. Raman spectra of selenium dioxide at high pressures. *Spectrosc. Lett.* **33**, 555-567 (2000).
9. Giannozzi, P. *et al.* Quantum ESPRESSO: a modular and open-source software project for quantum simulations of materials. *J. Phys. Condens. Matter* **21**, 395502 (2009).
10. Perdew, J. P., Burke, K., Ernzerhof, M. Generalized gradient approximation made simple. *Phys. Rev. Lett.* **77**, 3865 (1996).
11. Hamann, D. R. Optimized norm-conserving vanderbilt pseudopotentials. *Phys. Rev. B* **88**, 085117 (2013).
12. Baroni, S., de Gironcoli, S., Dal Corso, A. et al. Phonons and related crystal properties from density-functional perturbation theory. *Rev. Mod. Phys.* **73**, 515–562 (2001).
13. Kresse, G. & Hafner, J. Ab initio molecular dynamics for liquid metals. *Phys. Rev. B: Condens. Matter Mater. Phys.* **47**, 558-561 (1993).
14. Blöchl, P. E. Projector augmented-wave method. *Phys. Rev. B. Condens. Matter Mater. Phys.* **50**, 17953-17979 (1994).
15. Kresse, G. & Joubert, D. From Ultrasoft pseudopotentials to the projector augmented-wave method. *Phys. Rev. B: Condens. Matter Mater. Phys.* **59**, 1758-1775 (1999).
16. Perdew, J. P., Burke, L. & Ernzerhof, M. Generalized gradient approximation made simple. *Phys. Rev. Lett.* 77, 3865-3868 (1996).
17. Grimme, S. Semiempirical GGA-type density functional constructed with a long-range dispersion correction. *J. Comput. Chem.* 27, 1787-1799 (2000).
18. Mathew, K., Sundararaman, R., Letchworth-Weaver, K.; Arias, T. & Hennig, R. G. Implicit solvation model for density-functional study of nanocrystal surfaces and reaction pathways. *J. Chem. Phys.* **140**, 084106 (2014).
19. Mathew, K., Kolluru, V. C., Mula, S., Steinmann, S. N. & Hennig, R. G. Implicit self-consistent electrolyte model in plane-wave density-functional theory. *J. Chem. Phys.* **151**, 234101 (2019).
20. Henkelman, G., Uberuaga, B. P. & Jónsson, H. A climbing image nudged band method for finding saddle points and minimum energy paths. *J. Chem. Phys.* **113**, 9901-9904 (2000).
21. Nørskov, J. K. *et al*. Origin of the overpotential for oxygen reduction at a fuel-cell cathode. *J. Phys. Chem. B* **108**, 17886-17892 (2004).
22. Wang X. *et al*. Regulation of coordination number over single Co sites: triggering the efficient electroreduction of CO_2. *Angew. Chem. Int. Ed.* **57**, 1944-1948 (2018).
23. Duan, Z. & Henkelman, G. Theoretical resolution of the exceptional oxygen reduction activity of Au (100) in alkaline media. *ACS Catal.* **9**, 5567-5573 (2019).
24. Filhol, J. S. & Neurock, M. Elucidation of the electrochemical activation of water over Pd by first principles. *Angew. Chem. Int. Ed.* **118**, 416-420 (2006).
